# Supplementary material for: G-quadruplex DNA structures in human stem cells and differentiation
Source: Nat Commun. 2022 Jan 10;13:142. doi: 10.1038/s41467-021-27719-1 (PMC8748810; doi:10.1038/s41467-021-27719-1)
Supplement: Supplementary file 1 — Supplementary Information [file 41467_2021_27719_MOESM1_ESM.pdf]

G-quadruplex DNA structures in human stem cells and differentiation

Katherine G. Zyner[1,4], Angela Simeone[1,4], Sean M. Flynn[1], Colm Doyle[1], Giovanni Marsico[1], Santosh Adhikari[2], Guillem Portella[2], David Tannahill[1] and Shankar Balasubramanian[1,2,3,]

Affiliations:
[1]Cancer Research UK Cambridge Institute, Li Ka Shing Centre, Robinson Way, Cambridge, CB2 0RE, United Kingdom.
[2]Department of Chemistry, University of Cambridge, Lensfield Road, Cambridge CB2 1EW, United Kingdom.
[3]School of Clinical Medicine, University of Cambridge, Cambridge, CB2 0SP, United Kingdom.
[4]These authors contributed equally to this work.

*Correspondence to: sb10031@cam.ac.uk

Inventory of Supplementary Information:

Supplementary Figures (Supplementary Fig. 1 to 19)
Supplementary Discussion
Supplementary References

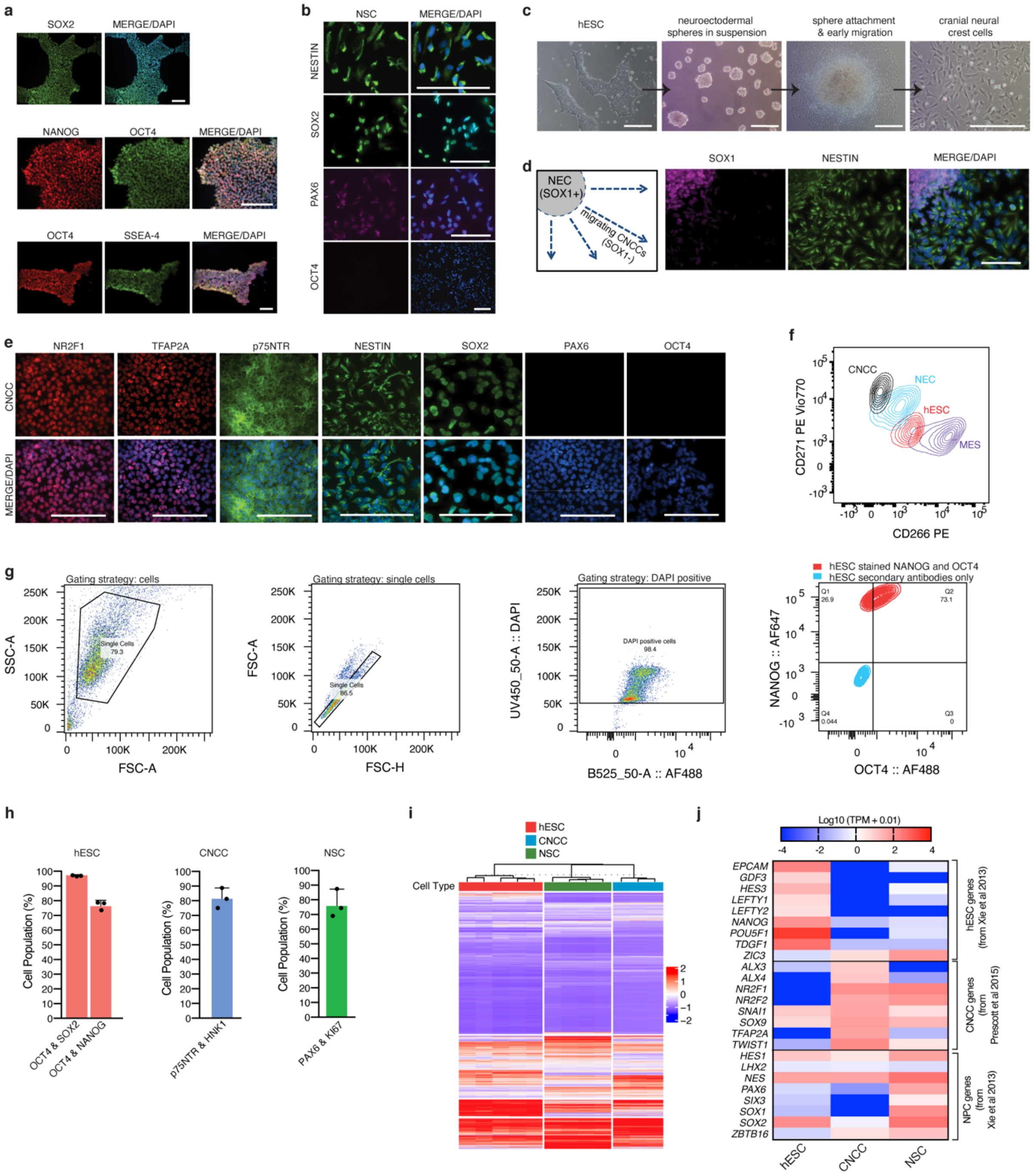
a
SOX2
MERGE/DAPI
NANOG
OCT4
MERGE/DAPI
OCT4
SSEA-4
MERGE/DAPI
b
NSC
MERGE/DAPI
NESTIN
SOX2
PAX6
OCT4
c
hESC
neuroectodermal spheres in suspension
sphere attachment & early migration
cranial neural crest cells
d
NEC (SOX1+)
migrating CNCCs (SOX1-)
SOX1
NESTIN
MERGE/DAPI
e
NR2F1
TFAP2A
p75NTR
NESTIN
SOX2
PAX6
OCT4
CNCC
MERGE/DAPI
f
CNCC
NEC
hESC
MES
CD271 PE Vio770
CD266 PE
g
Gating strategy: cells
Single Cells 79.3
SSC-A
FSC-A
Gating strategy: single cells
Single Cells 86.5
FSC-A
FSC-H
Gating strategy: DAPI positive
DAPI positive cells 98.4
UV450_50-A :: DAPI
B525_50-A :: AF488
hESC stained NANOG and OCT4
hESC secondary antibodies only
NANOG :: AF647
OCT4 :: AF488
h
hESC
CNCC
NSC
Cell Population (%)
OCT4 & SOX2
OCT4 & NANOG
p75NTR & HNK1
PAX6 & KI67
i
hESC
CNCC
NSC
Cell Type
j
Log10 (TPM + 0.01)
EPCAM
GDF3
HES3
LEFTY1
LEFTY2
NANOG
POU5F1
TDGF1
ZIC3
ALX3
ALX4
NR2F1
NR2F2
SNAI1
SOX9
TFAP2A
TWIST1
HES1
LHX2
NES
PAX6
SIX3
SOX1
SOX2
ZBTB16
hESC genes (from Xie et al 2013)
CNCC genes (from Prescott et al 2015)
NPC genes (from Xie et al 2013)
hESC
CNCC
NSC

Supplementary Figure 1: Characterisation of human stem cell types. a-b, Representative immunofluorescent microscopy (IF) images of (a) hESCs and (b) NSCs stained for hESC markers: OCT4, NANOG, SOX2 and SSEA-4 (OCT4, NANOG IF data are also shown in Fig 1.c) and known NSC markers: SOX2, NESTIN and PAX6 (NESTIN and PAX6 IF data are also shown in Fig 1c). Scale bar = 100µm. **c,** Brightfield images of steps in derivation of CNCCs: hESCs are first differentiated into neuroectodermal spheres (NEC) which are then allowed to attach for the generation of migratory CNCCs. Scale bar = 250µm. **d,** Representative IF images showing NESTIN+/SOX1- (neural crest) cells migrating out from NESTIN+/SOX1+ neural rosettes. Scale bar = 100µm. **e,** Representative IF images of CNCCs stained with antibodies against neural crest markers NR2F1, TFAP2A, p75NTR, NESTIN, SOX2; hESC marker OCT4 and NSC marker PAX6 (TFAP2A and p75NTR IF data are also shown in Fig 1c). Scale bar = 100µm. **f,** Flow cytometry plots for cell surface markers CD271 and CD266 during derivation of CNCCs from hESCs, as well as further differentiation to mesenchymal lineages (in agreement with Prescott 2015[1]). **g,** Example of gating strategy used for determining percentage stem cells positive for tested cell lineage markers (e.g., hESCs positive for NANOG and OCT4) via flow cytometry. **h,** Percentage of hESCs positive for OCT4 and NANOG or OCT4 and SOX2; NSCs positive for PAX6 and Ki67 and CNCCs positive for neural crest markers p75NTR and HNK1 via flow cytometry. Mean ± SD from 3 independent biological replicates. **i,** Hierarchical clustering of genes based on z-score of expression values (median TPM). Row: genes, columns: individual stem cell libraries. **j,** Heatmap showing median gene expression (Log10(TPM + 0.01)) for the indicated genes for hESCs[2], CNCCs[1] and neural progenitor cells (NPC) developmentally analogous to NSCs[2] for each stem cell line.

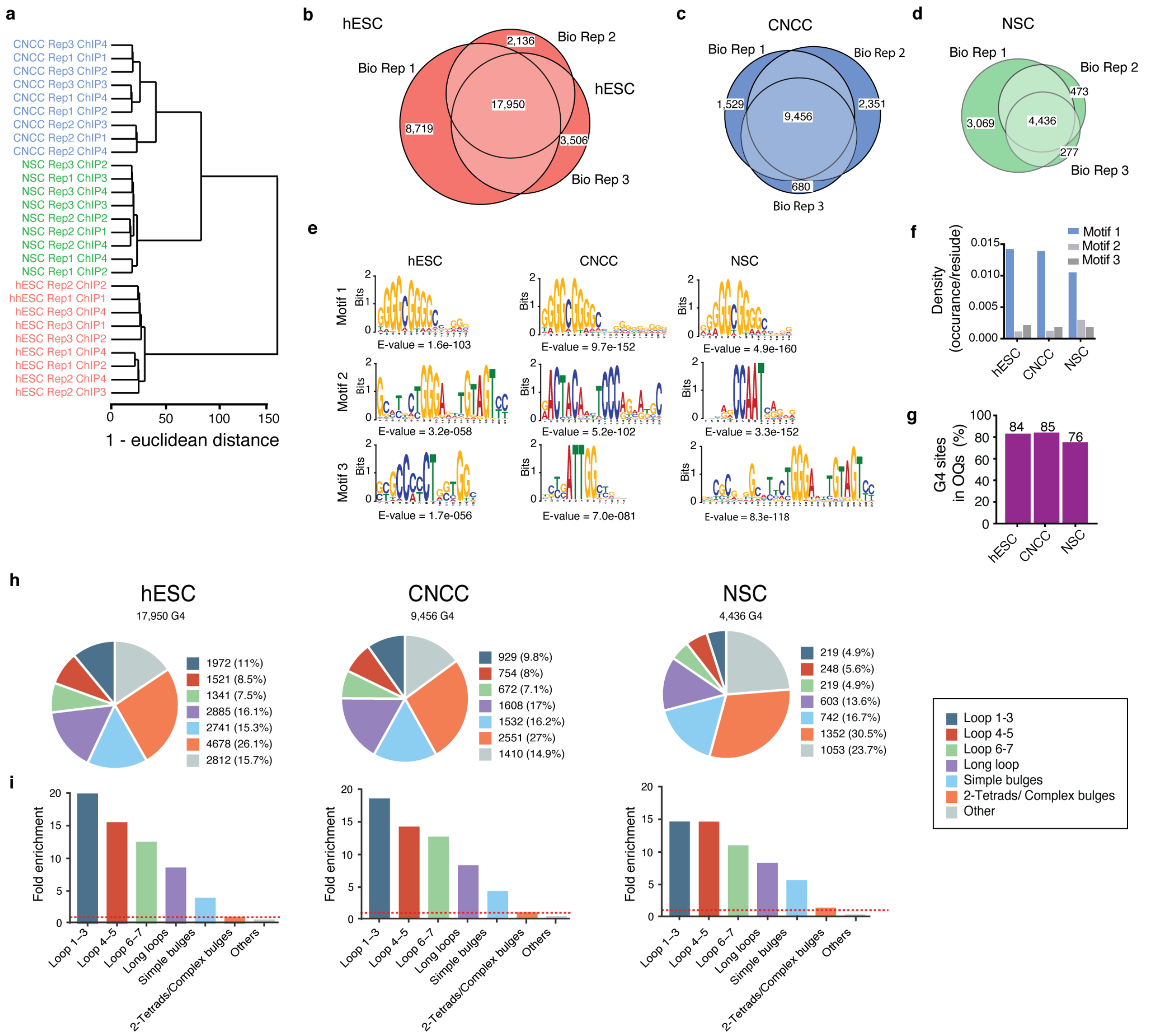

Supplementary Figure 2: Quality control for G4 ChIP-seq. a, Hierarchical clustering of reads per million (RPM) for G4s (G4 ChIP-seq libraries) generated for 3 biological replicates (i.e., Rep) with 3 technical replicates (i.e., ChIP) each for stem cell type. **b-d,** Area proportional Venn diagrams of G4 ChIP-seq peaks called by macs2 for (b) hESCs, (c) CNCCs and (d) NSCs for 3 biological replicates. Values given in the center correspond to the number of G4 ChIP-seq peaks in common for at least 2 out of 3 biological replicates, hereinafter called G4s. **e,** Top 3 sequence motifs identified by Multiple Em for motif elicitation (MEME)-ChIP[3] in G4s for each stem cell type. **f,** Motif density (motif occurrence per residue) in G4s calculated via Find individual motif occurrences (FIMO)[4]. Only the G-rich motif showed an increase in density for G4s relative to other motifs. **g,** Percentage of G4s identified in sequences with the ability to fold into a G4 *in vitro* (observed quadruplex sequences; OQs)[5]. **h,** Enrichment analysis for different G4 structural classes. The total number of G4 regions in each structural class, per stem cell line are shown. Loop size 1–3, 4–5 and 6– 7, indicates that at least one loop of this length is present in the G4; long loop indicates a G4 with any loop of length >7 (up to 12 for any loop and 21 for the middle loop); simple bulge indicates a G4 with a bulge of 1–7 bases in one G-run or multiple 1-base bulges; 2-tetrads / complex bulge indicates G4s with two G-bases per G-run or several bulges of 1–5 bases; and other indicates other G4-types that do not fall into the former categories. **i,** Fold enrichment for each structural class in (g) compared to random (average of 3 randomisations). Higher enrichment values equate to the higher likelihood of being present among the G4 regions.

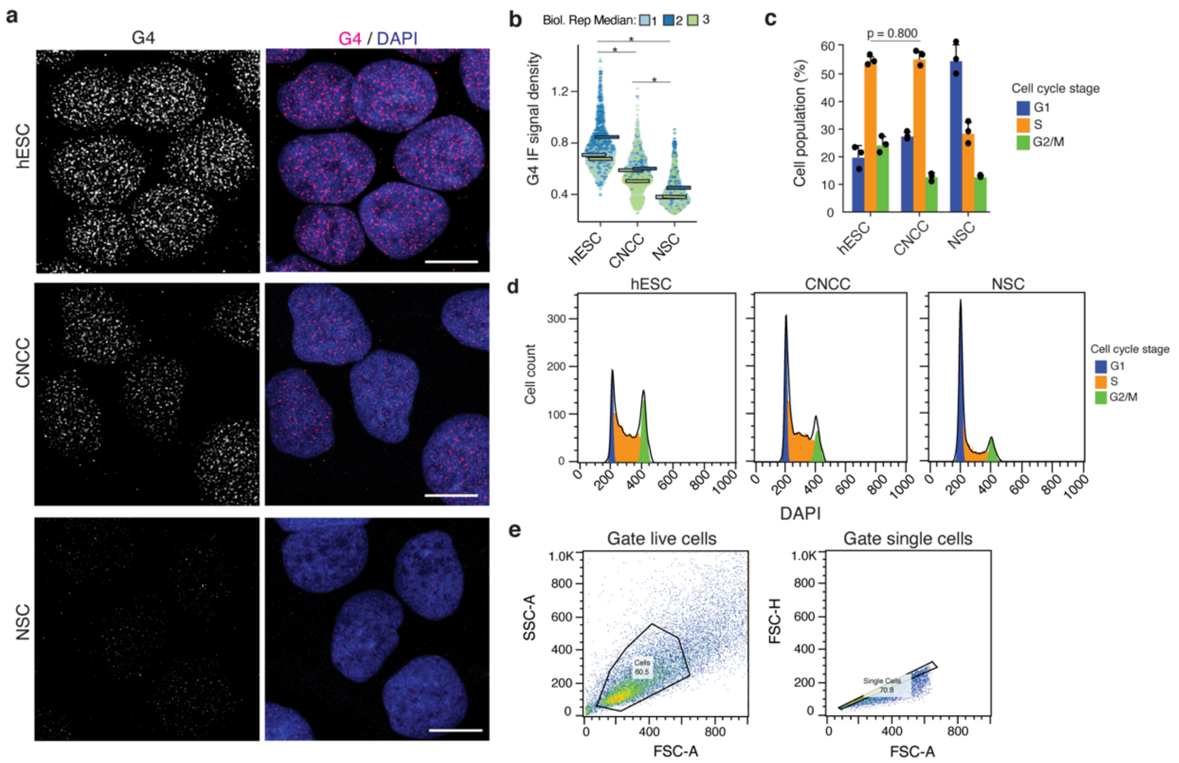

Supplementary Figure 3: G4 abundance decreases with loss of stem cell potency. a, Representative immunofluorescent microscopy images for hESCs, CNCCs and NSCs stained with the G4 structure-specific antibody BG4[6] after pre-treatment with RNase A. Central slice of 13 slice confocal Z-stack (captured 0.3 µm apart) is shown. Left panels show G4 foci and right panels show G4 foci together with the nuclear counterstain DAPI (blue). Scale bar is 10 µm. **b,** Quantification of the total G4 signal density (sum G4 signal /sum DAPI signal) per nucleus calculated on the sum projection of the Z-stack. Each point represents a single nucleus, and the coloured horizontal bars represent the median from each biological replicate. N = 3 biologically independent experiments. Exact number of cells can be found in Source Data. *: p < 2E-16, one-sided Kolmogorov-Simonov test, per biological replicate. **c-d**, Cell cycle analysis of stem cell populations as determined by DAPI staining and flow cytometry. Quantification of the proportion of the cells in each phase of the cell cycle presented in (c) and representative cell cycle profiles are shown in (d). Bar graph shows Mean ± SD. N = 3 independent biological replicates of 10,000 cells each. Statistical test: one-sided Mann-Whitney U test. **e,** Example of flow cytometry gating strategy used to generate histograms in (d).

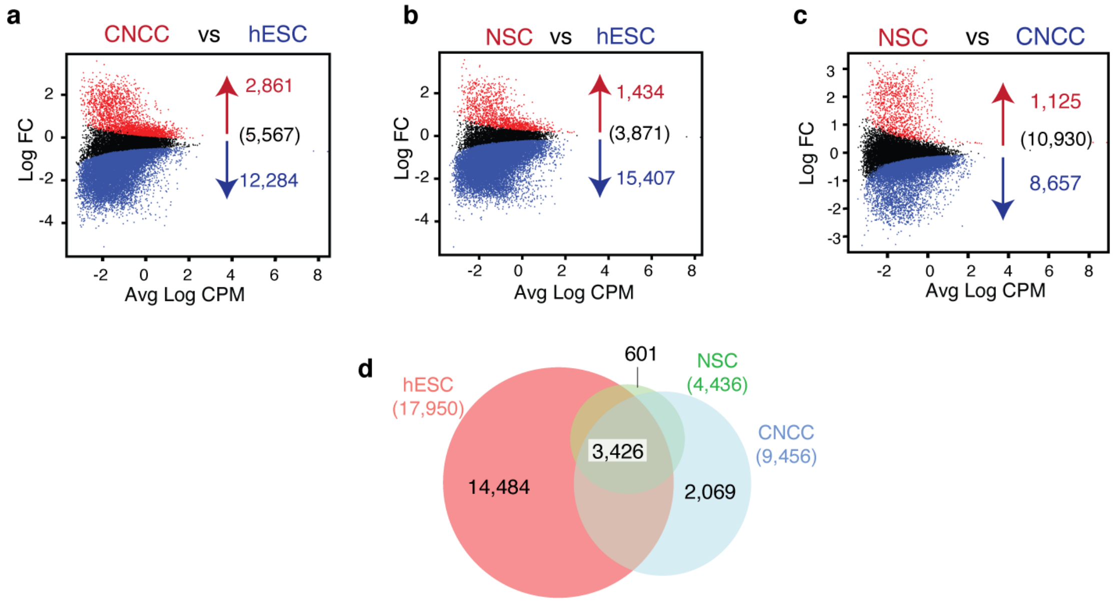

Supplementary Figure 4: Most CNCC and NSC G4s are found in hESC. a-c, MA plot showing differential binding analysis across 20,712 sites showing significant (FDR < 0.05) differences in G4 signal intensities between (a) hESCs and CNCCs, (b) hESCs and NSCs or (c) CNCCs and NSCs (log_2fold change vs log_2 counts per million). 66% (5,567/8,428) of CNCC and 73% (3,871/ 5,305) of NSC G4s are in common with hESCs. 91% (10,930/12,055) of NSC G4s are in common with CNCCs. **d**, Intersection of G4s between all three stem cell types. Only G4s common to all three stem cells or unique to each stem cell type are shown.

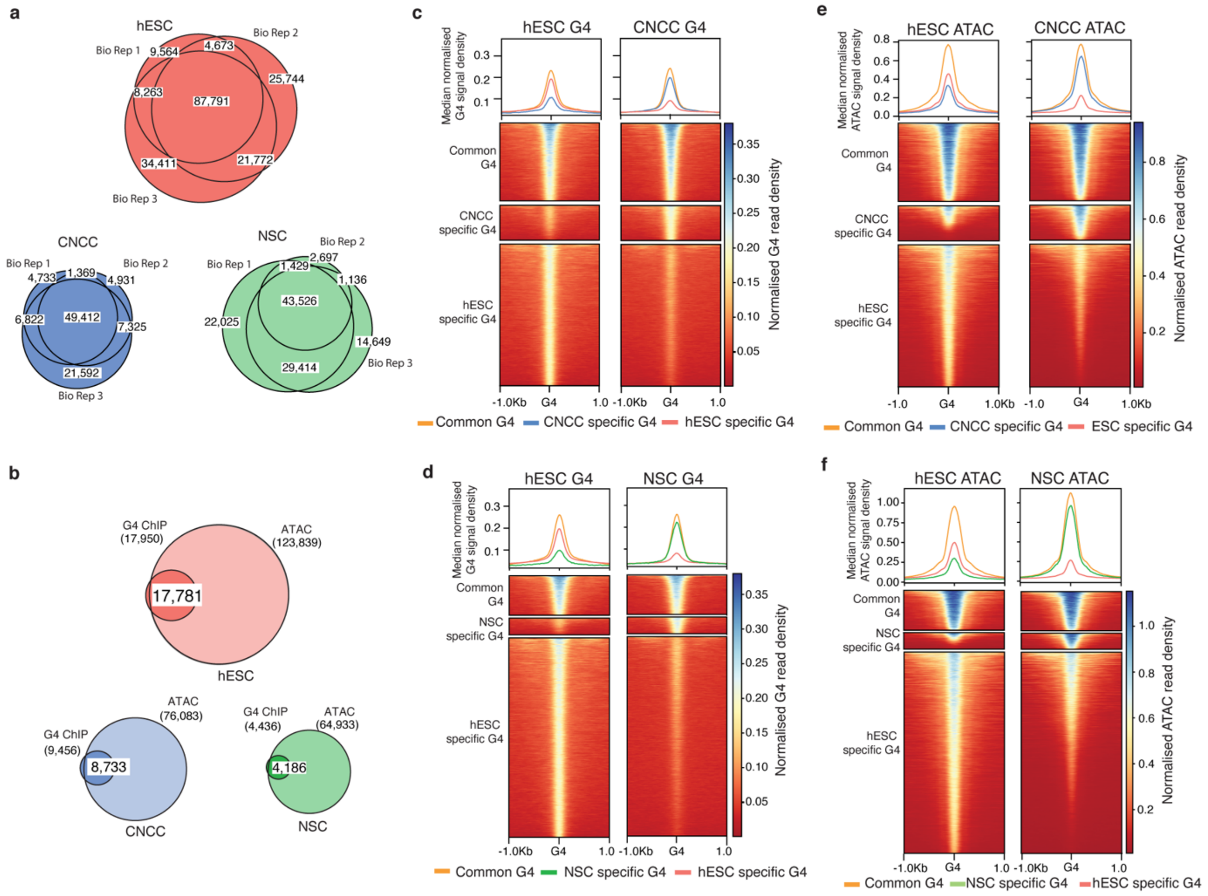

Supplementary Figure 5: G4 loss is linked to reduced chromatin accessibility during differentiation. a-b, Area proportional Venn diagrams of (a) ATAC-seq peaks (ATAC) called by macs2 for hESCs, CNCCs and NSCs for 3 biological replicates and (b) G4s overlapping ATAC sites in each stem cell type. **c-d,** Heatmaps showing normalised G4 read density across common or cell type-specific G4 sites derived for pairwise comparisons between (c) hESCs and CNCCs and (d) hESCs and NSCs. **e-f**, Heatmaps showing normalised ATAC-seq read density across common or cell type specific G4s derived from pairwise comparisons between (e) hESCs and CNCCs and (f) hESCs and NSCs.

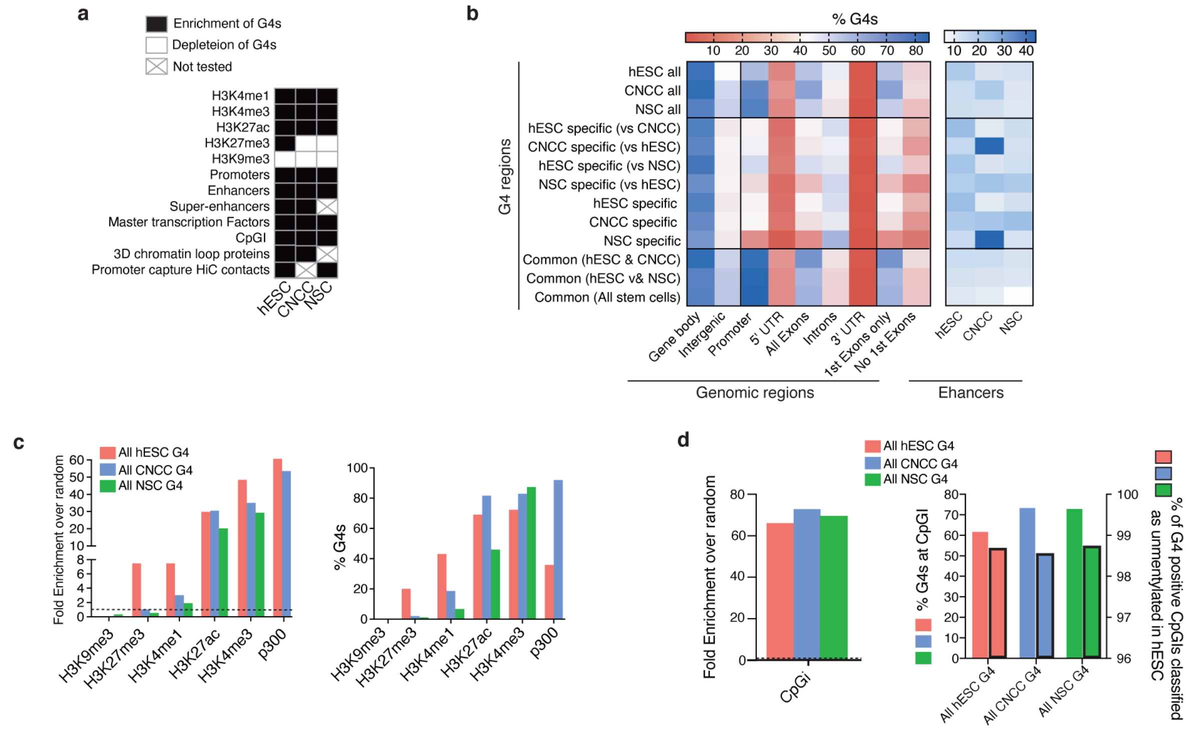

Supplementary Figure 6: G4s are found in stem cell regulatory elements. a, Summary table of genomic and epigenomic features compared to G4 presence. **b,** Proportion of G4s found at genomic features, as defined in Fig.1 (TSS, transcription start site), and enhancers. Rows; G4s and columns; genomic and epigenomic features. **c-d**, Fold-enrichments over random (n = 1,000 permutations) and proportion of G4s per stem cell type at (c) the histone marks and histone acetyltransferase (p300); and (d) CpG islands (CpGi, tracks from USCS genome browser). hESC hypomethylated CpG islands are defined as in Lee *et al* 2017[7].

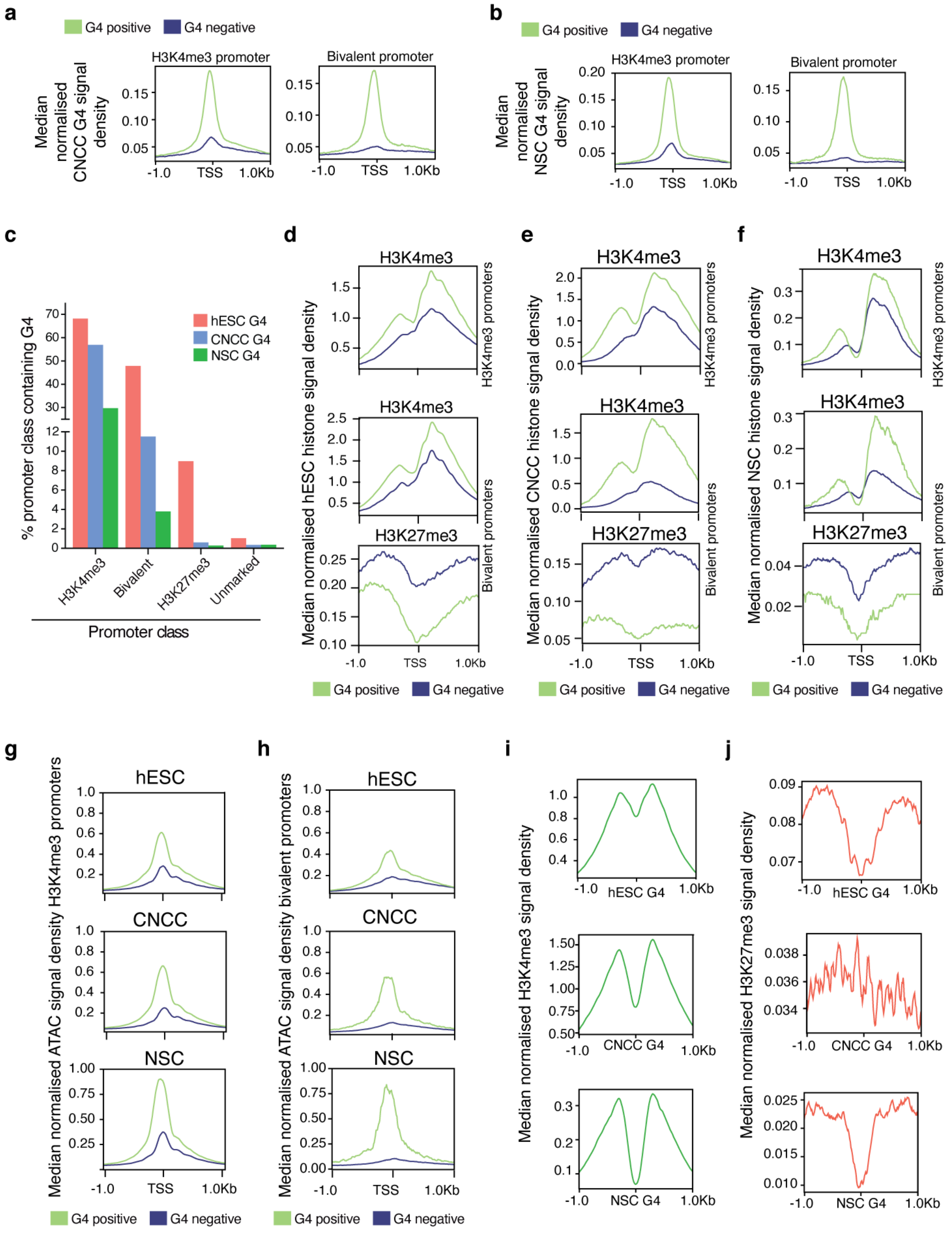

a
G4 positive
G4 negative
H3K4me3 promoter
Bivalent promoter
Median normalised CNCC G4 signal density
b
NSC G4 signal density
c
% promoter class containing G4
hESC G4
CNCC G4
NSC G4
H3K4me3
Bivalent
H3K27me3
Unmarked
Promoter class
d
Median normalised hESC histone signal density
H3K4me3 promoters
Bivalent promoters
e
Median normalised CNCC histone signal density
f
Median normalised NSC histone signal density
g
Median normalised ATAC signal density H3K4me3 promoters
hESC
CNCC
NSC
h
Median normalised ATAC signal density bivalent promoters
i
Median normalised H3K4me3 signal density
hESC G4
CNCC G4
NSC G4
j
Median normalised H3K27me3 signal density

Supplementary Figure 7: G4s are a structural feature of bivalent and H3K4me3 promoters. a-b, Median normalised (a) CNCC or (b) NSC G4 ChIP-seq signal density at H3K4me3 and bivalent promoter regions. TSS, transcription start site. **c,** Proportion of all hESC, CNCC or NSC G4s overlapping histone modification promoter classes. **d-f,** Median normalised H3K4me3 read density across (d) hESC (also presented in Fig. 3), (e) CNCC and (f) NSC H3K4me3 promoters (top) or H3K4me3 and H3K4me27 read density across bivalent promoter regions (bottom) segregated by presence (green) or absence (dark blue) of G4. **g-h,** Median normalised ATAC-seq signal at (g) H3K4me3 and (h) bivalent promoters in hESCs (top, also presented in Fig. 3), CNCCs (middle) or NSCs (bottom). **i-j,** Median normalised ChIP-seq signal density of H3K4me3 (i) and H3K27me3 (j) at hESCs (top), CNCCs (middle) and NSCs (bottom) promoters centered around G4 sites.

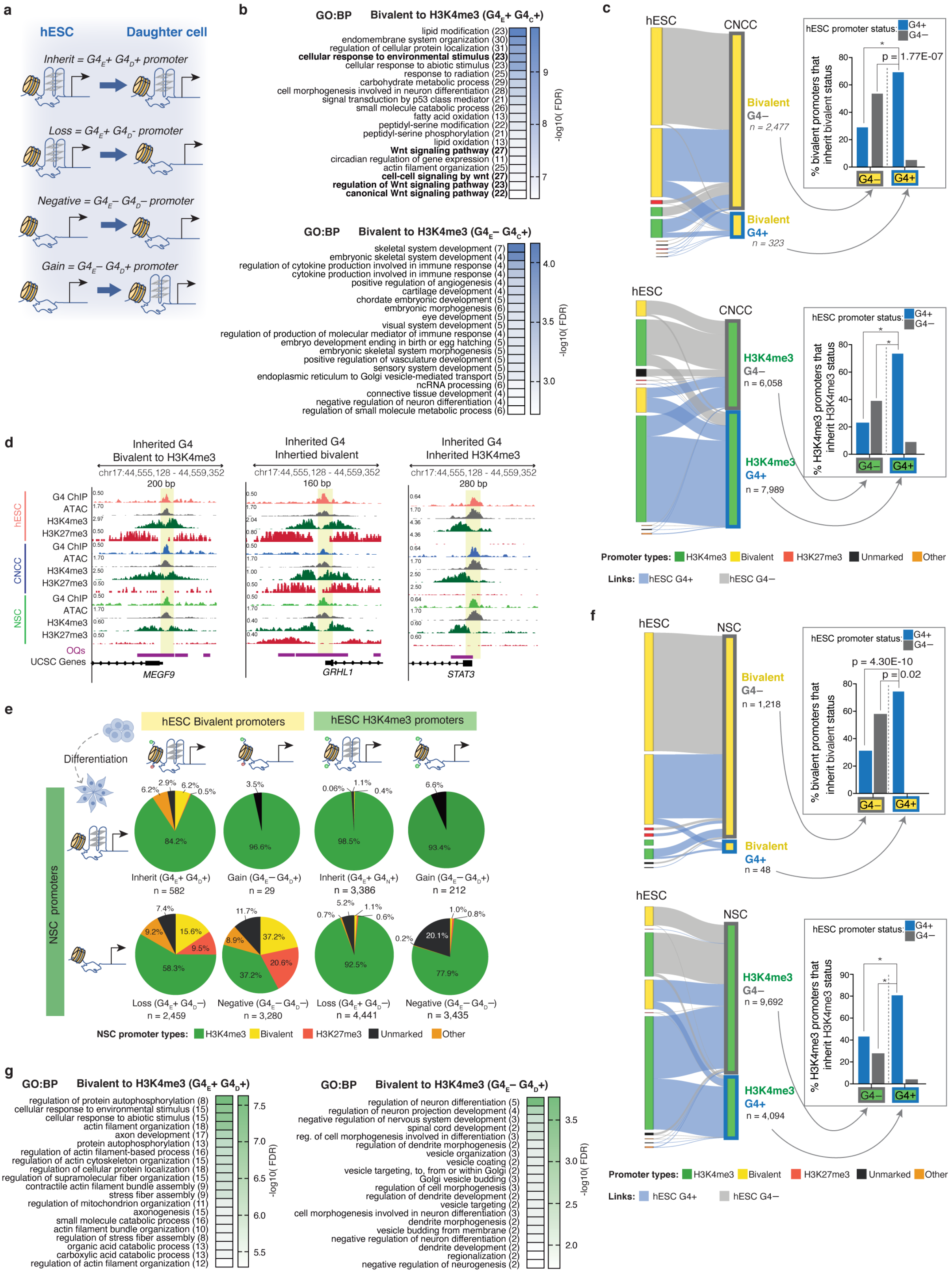
a
hESC
Daughter cell
Inherit = G4E+ G4D+ promoter
Loss = G4E+ G4D- promoter
Negative = G4E– G4D– promoter
Gain = G4E– G4D+ promoter
b
GO:BP Bivalent to H3K4me3 (G4E+ G4C+)
lipid modification (23)
endomembrane system organization (30)
regulation of cellular protein localization (31)
cellular response to environmental stimulus (23)
cellular response to abiotic stimulus (23)
response to radiation (25)
carbohydrate metabolic process (29)
cell morphogenesis involved in neuron differentiation (28)
signal transduction by p53 class mediator (21)
small molecule catabolic process (26)
fatty acid oxidation (13)
peptidyl-serine modification (22)
peptidyl-serine phosphorylation (21)
lipid oxidation (13)
Wnt signaling pathway (27)
circadian regulation of gene expression (11)
actin filament organization (25)
cell-cell signaling by wnt (27)
regulation of Wnt signaling pathway (23)
canonical Wnt signaling pathway (22)
-log10(FDR)
GO:BP Bivalent to H3K4me3 (G4E– G4C+)
skeletal system development (7)
embryonic skeletal system development (4)
regulation of cytokine production involved in immune response (4)
cytokine production involved in immune response (4)
positive regulation of angiogenesis (4)
cartilage development (4)
chordate embryonic development (5)
embryonic morphogenesis (6)
eye development (5)
visual system development (5)
regulation of production of molecular mediator of immune response (4)
embryo development ending in birth or egg hatching (5)
embryonic skeletal system morphogenesis (5)
positive regulation of vasculature development (5)
sensory system development (5)
endoplasmic reticulum to Golgi vesicle-mediated transport (5)
ncRNA processing (6)
connective tissue development (4)
negative regulation of neuron differentiation (4)
regulation of small molecule metabolic process (6)
c
hESC
CNCC
hESC promoter status: G4+ G4–
p = 1.77E-07
% bivalent promoters that inherit bivalent status
Bivalent G4– n = 2,477
Bivalent G4+ n = 323
H3K4me3 G4– n = 6,058
H3K4me3 G4+ n = 7,989
% H3K4me3 promoters that inherit H3K4me3 status
Promoter types: H3K4me3 Bivalent H3K27me3 Unmarked Other
Links: hESC G4+ hESC G4–
d
Inherited G4 Bivalent to H3K4me3
Inherited G4 Inhertied bivalent
Inherited G4 Inherited H3K4me3
chr17:44,555,128 - 44,559,352
200 bp
160 bp
280 bp
hESC
CNCC
NSC
G4 ChIP
ATAC
H3K4me3
H3K27me3
OQs
UCSC Genes
MEGF9
GRHL1
STAT3
e
Differentiation
hESC Bivalent promoters
hESC H3K4me3 promoters
NSC promoters
Inherit (G4E+ G4D+) n = 582
Gain (G4E– G4D+) n = 29
Inherit (G4E+ G4N+) n = 3,386
Gain (G4E– G4D+) n = 212
Loss (G4E+ G4D–) n = 2,459
Negative (G4E– G4D–) n = 3,280
Loss (G4E+ G4D–) n = 4,441
Negative (G4E– G4D–) n = 3,435
NSC promoter types: H3K4me3 Bivalent H3K27me3 Unmarked Other
f
hESC
NSC
p = 4.30E-10
p = 0.02
Bivalent G4– n = 1,218
Bivalent G4+ n = 48
H3K4me3 G4– n = 9,692
H3K4me3 G4+ n = 4,094
Promoter types: H3K4me3 Bivalent H3K27me3 Unmarked Other
Links: hESC G4+ hESC G4–
g
GO:BP Bivalent to H3K4me3 (G4E+ G4D+)
regulation of protein autophosphorylation (8)
cellular response to environmental stimulus (15)
cellular response to abiotic stimulus (15)
actin filament organization (18)
axon development (17)
protein autophosphorylation (13)
regulation of actin filament-based process (16)
regulation of actin cytoskeleton organization (15)
regulation of cellular protein localization (18)
regulation of supramolecular fiber organization (15)
contractile actin filament bundle assembly (9)
stress fiber assembly (9)
regulation of mitochondrion organization (11)
axonogenesis (15)
small molecule catabolic process (16)
actin filament bundle organization (10)
regulation of stress fiber assembly (8)
organic acid catabolic process (13)
carboxylic acid catabolic process (13)
regulation of actin filament organization (12)
GO:BP Bivalent to H3K4me3 (G4E– G4D+)
regulation of neuron differentiation (5)
regulation of neuron projection development (4)
negative regulation of nervous system development (3)
spinal cord development (2)
reg. of cell morphogenesis involved in differentiation (3)
regulation of dendrite morphogenesis (2)
vesicle organization (3)
vesicle coating (2)
vesicle targeting, to, from or within Golgi (2)
Golgi vesicle budding (3)
regulation of cell morphogenesis (3)
regulation of dendrite development (2)
vesicle targeting (2)
cell morphogenesis involved in neuron differentiation (3)
dendrite morphogenesis (2)
vesicle budding from membrane (2)
negative regulation of neuron differentiation (2)
dendrite development (2)
regionalization (2)
negative regulation of neurogenesis (2)

Supplementary Figure 8: G4s are associated with hESC H3K4me3 and bivalent promoters during differentiation. a, Schematic of G4 promoter signature: $G4_E+$ $G4_D+$: G4 inheritance from hESCs to daughter cells; $G4_E+$ $G4_D-$: G4 lost in daughter cells; $G4_E-$ $G4_D+$: G4 gained in the daughter cells and $G4_E-$ $G4_D-$: promoters lacking a G4s in both stem cells. **b,** Top 20 significant (FDR <0.05) Gene Ontology biological processes (GO: BP) obtained using g:Profiler, for genes with promoters that transition from bivalent to H3K4me3 and maintain or gain a G4 after differentiation of hESCs into CNCCs. The number of genes intersected is shown in brackets. Terms in bold are associated with Wnt signalling pathways. **c,** Alluvial plots showing origin of all CNCC bivalent (top) and H3K4me3 (bottom) promoters, classified by G4 presence or absence. Top inset, proportion of bivalent CNCC promoters that originate from hESC bivalent promoters, classified by G4 presence or absence. Bottom inset, similar quantification for CNCC H3K4me3 promoters. See Source Data for further details. *: $p< 2E-16$, one-sided Pearson's χ^2 test for proportions. **d,** Genome browser view for representative gene promoters demonstrating maintenance of G4 leading to transition from bivalent to H3K4me3 status (*MEGF*), maintenance of bivalent status (*GRHL1*) or maintenance of H3K4me3 status (*STAT3*) after hESC differentiation into CNCCs or NSCs. Yellow box highlights overlap of G4s, open chromatin sites (ATAC) and observed quadruplex sequences, (OQs: G4s confirmed to fold i*n vitro*)[5]. **e,** Pie charts showing histone modification transitions for bivalent and H3K4me3 hESC promoters after differentiation to NSCs, segregated by G4 promoter signature. **f,** Same analysis as described in (b) for hESC to NSC transition**. g,** Same analysis as described in C for hESC to NSC transitions. See Source Data for further details. *: $p< 2E-16$, one-sided Pearson's χ^2 test for proportions.

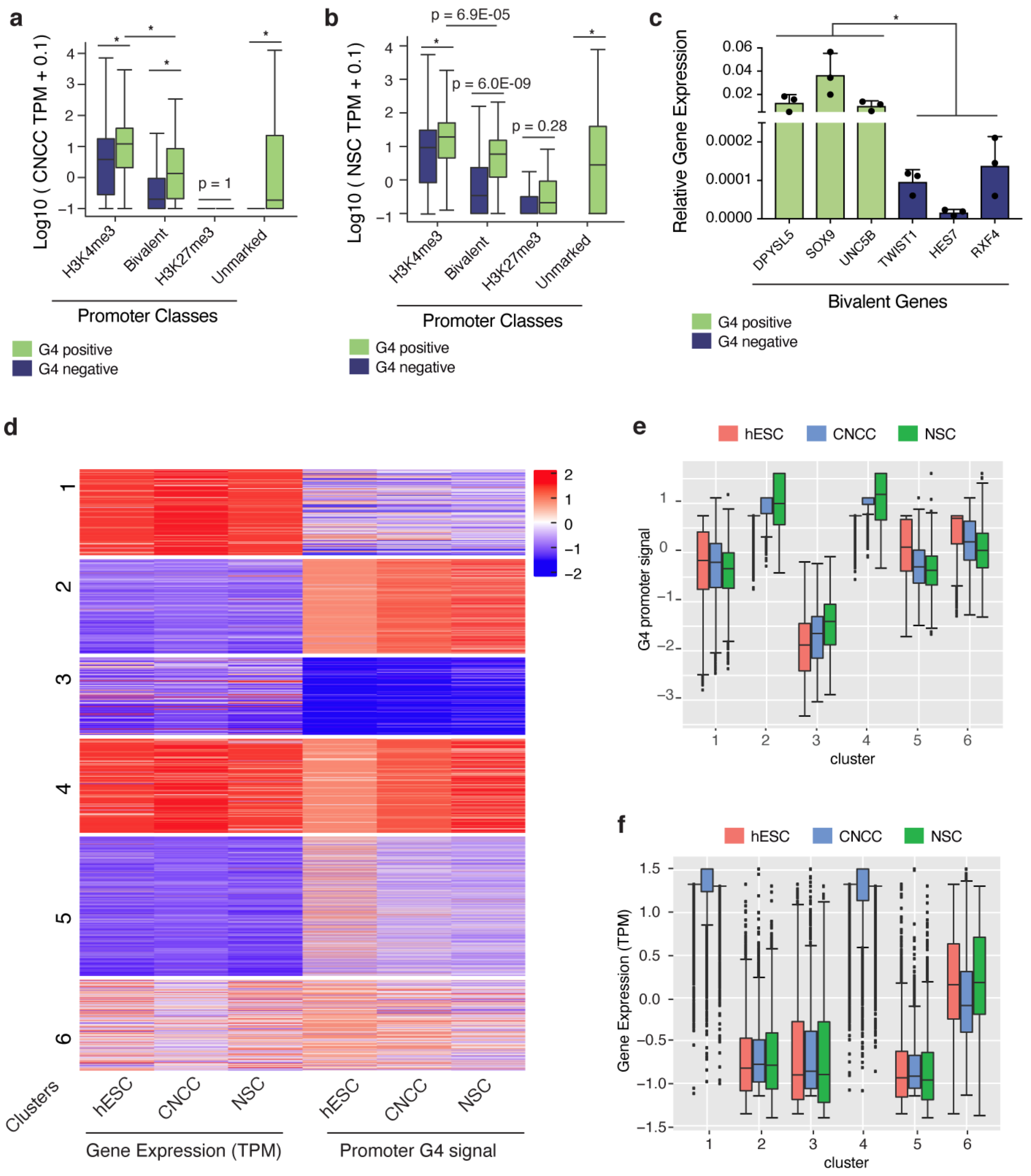
a
Log10 (CNCC TPM + 0.1)
H3K4me3
Bivalent
H3K27me3
Unmarked
p = 1
Promoter Classes
G4 positive
G4 negative
b
p = 6.9E-05
p = 6.0E-09
p = 0.28
Log10 (NSC TPM + 0.1)
H3K4me3
Bivalent
H3K27me3
Unmarked
Promoter Classes
G4 positive
G4 negative
c
Relative Gene Expression
DPYSL5
SOX9
UNC5B
TWIST1
HES7
RXF4
Bivalent Genes
G4 positive
G4 negative
d
Clusters
hESC
CNCC
NSC
hESC
CNCC
NSC
Gene Expression (TPM)
Promoter G4 signal
e
hESC
CNCC
NSC
G4 promoter signal
cluster
f
hESC
CNCC
NSC
Gene Expression (TPM)
cluster

Supplementary Figure 9: Promoter G4s and gene expression. a-b, Gene expression levels (log10[average TPM + 0.1], transcript per million averaged across biological replicates) for (a) CNCCs or (b) NSCs. Histone modification promoter classes as defined in Fig. 2B. G4-positive promoters (green) and G4-negative (dark blue). Data are presented as median (centre) and interquartile range (box; the lower and upper bounds of the box represent the 25th and 75th percentiles, respectively). Whiskers represent ± 1.5x interquartile range. N = 3 and 4 biologically independent samples for CNCCs and NSCs, respectively. Number of genes per group: CNCC G4 positive (H3K4me3: 7989, bivalent: 323, H3K27me3: 10 and unmarked: 135), CNCC G4 negative (H3K4me3: 6058, bivalent: 2477, H3K27me3: 1657 and unmarked: 39347), NSC G4 positive (H3K4me3: 4094, bivalent: 48, H3K27me3: 4 and unmarked: 150), NSC G4 negative (H3K4me3: 9692, bivalent: 1218, H3K27me3: 1420 and unmarked: 41147). *p < 2E-16, one-sided Kolmogorov-Smirnov test. **c,** Relative expression levels (normalised to *GAPDH*) of hESC genes with bivalent promoters that are G4 positive (green) or G4 negative (dark blue). Mean ± SD of N = 3 independent biological samples. *: p< 0.05 (unpaired two-sided t-test, see Source Data). **d,** K-medoid clustering (K=6) of genes based on z-score of expression level (median TPM) and G4 promoter signal; rows: promoters (n = 11,876), columns: individual stem cell types. N = 5, 3 and 4 biologically independent samples for hESCs, CNCCs and NSCs, respectively. Number of genes per cluster: cluster 1 (1754), cluster 2 (1929), cluster 3 (1577), cluster 4 (1920), cluster 5 (2846) and cluster 6 (1850). **e-f**, Box and whisker plots showing (e) z-score of G4 promoter signal and (f) z-score of gene transcription level for genes in each of the 6 clusters identified in (d). Data are presented as median (centre) and interquartile range (box; the lower and upper bounds of the box represent the 25th and 75th percentiles, respectively). Whiskers represent ± 1.5x interquartile range. See Source Data.

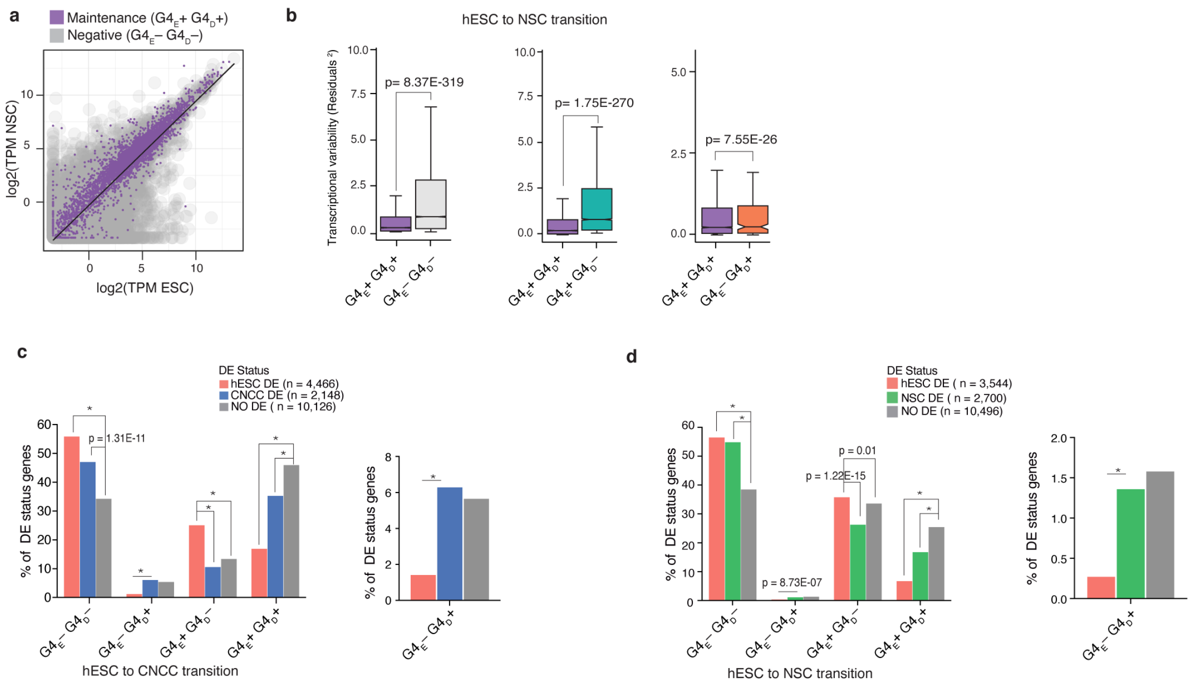

Supplementary Figure 10: G4s and differential gene expression changes during differentiation. a, Scatter plot of the log2 median transcripts per million (TPM) for hESCs (x-axis) vs NSCs (y-axis) with fitted weighted linear regression model (black line). $G4_E$; G4 promoter status in hESCs and $G4_D$; G4 promoter status in daughter cell (NSCs). **b,** Box plots of the calculated residuals of $G4_E$+ G4 $_D$+ and $G4_E$– G4 $_D$–, $G4_E$+ G4 $_D$– or $G4_E$– G4 $_D$+. Data are presented as median (centre) and interquartile range (box; the lower and upper bounds of the box represent the 25th and 75th percentiles, respectively). Whiskers represent ± 1.5x interquartile range. N = 5 and 4 biologically independent samples for hESCs and NSCs, respectively. Number of genes per group: $G4_E$+ $G4_D$+ (3849) and $G4_E$– $G4_D$– (18698), $G4_E$+ $G4_D$– (6838) or $G4_E$– $G4_D$+ (254). One-tailed F- test for variances. **c-d**, Proportion of differentially expressed (DE) genes categorised by G4 promoter signature for the comparison between (c) hESCs vs CNCCs and (d) hESCs vs NSCs. For each cell pair, genes were stratified into three groups: DE in hESCs (Log2FC < -1, FDR < 0.05), DE in the daughter cell (Log2FC > 1, FDR < 0.05) and not DE (NO DE). N = 5, 3 and 4 biologically independent samples for hESCs, CNCCs and NSCs, respectively. See Source Data for number of genes per group. *: p< 2E-16; one-sided Pearson's χ^2 test for proportions. Right graph in (c) and (d) is a zoomed in view for $G4_E$– $G4_D$+.

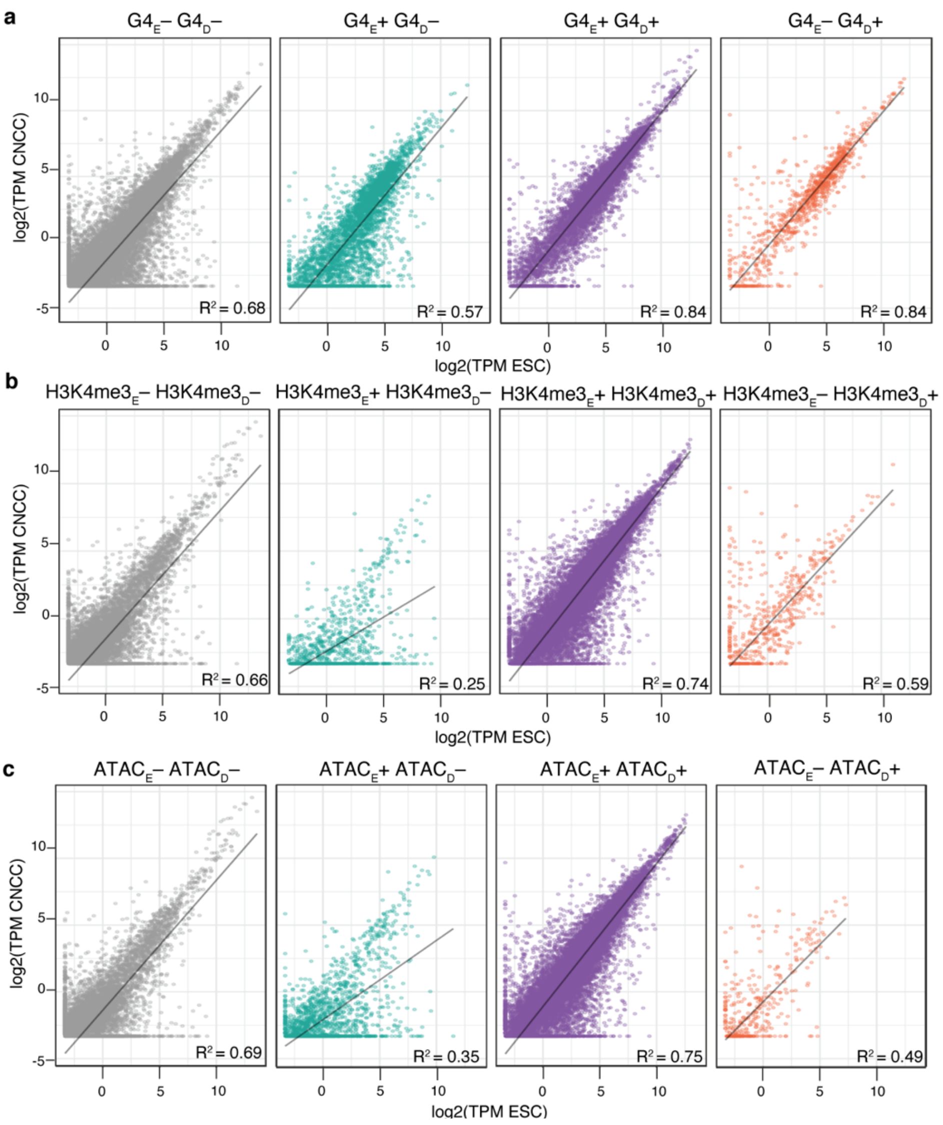

Supplementary Figure 11: Maintenance of G4s, ATAC or H3K4me3 promoter status is linked to transcriptional stabilisation during hESC to CNCC differentiation. a-c, Scatter plots of median transcripts per million (TPM) for hESCs vs CNCCs for genes categorised by (a) G4s, (b) H3K4me3 or (c) open chromatin (defined by ATAC-seq, ATAC) promoter classification. For each pairwise analysis, a weighted linear regression model was fitted to gene expression values (grey line) and the residuals computed to quantify transcriptional variation (see Methods). The coefficient of determination and goodness of fit, R^2 are displayed in the bottom right-hand corner.

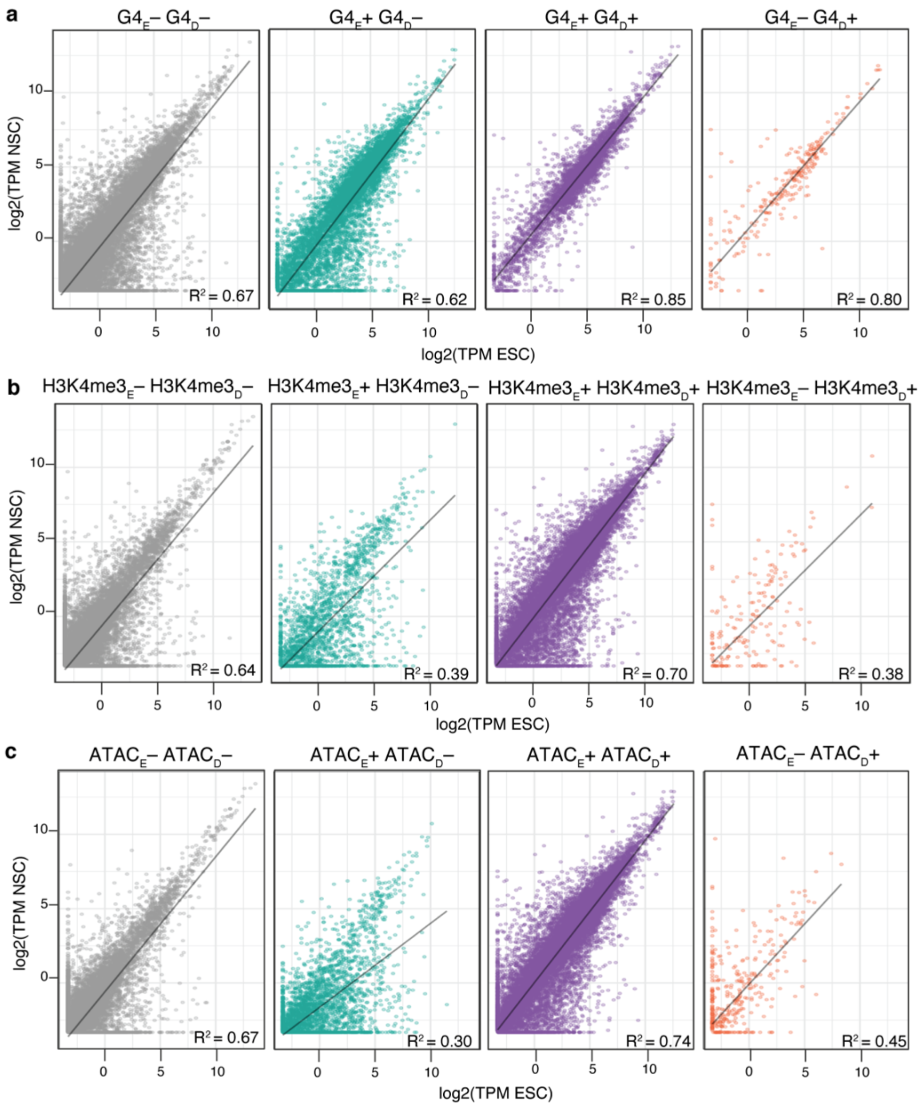

Supplementary Figure 12: Maintenance of G4s, ATAC or H3K4me3 promoter status is linked to transcriptional stabilisation during hESC to NSC differentiation. a-c, Scatter plots of median transcripts per million (TPM) for hESCs vs NSCs for genes categorised by (a) G4s, (b) H3K4me3 or (c) open chromatin (defined by ATAC-seq, ATAC) promoter classification. For each pairwise analysis, a weighted linear regression model was fitted to gene expression values (grey line) and the residuals computed to quantify transcriptional variation (see Methods). The coefficient of determination and goodness of fit, $R^{2,}$ are displayed in the bottom right-hand corner.

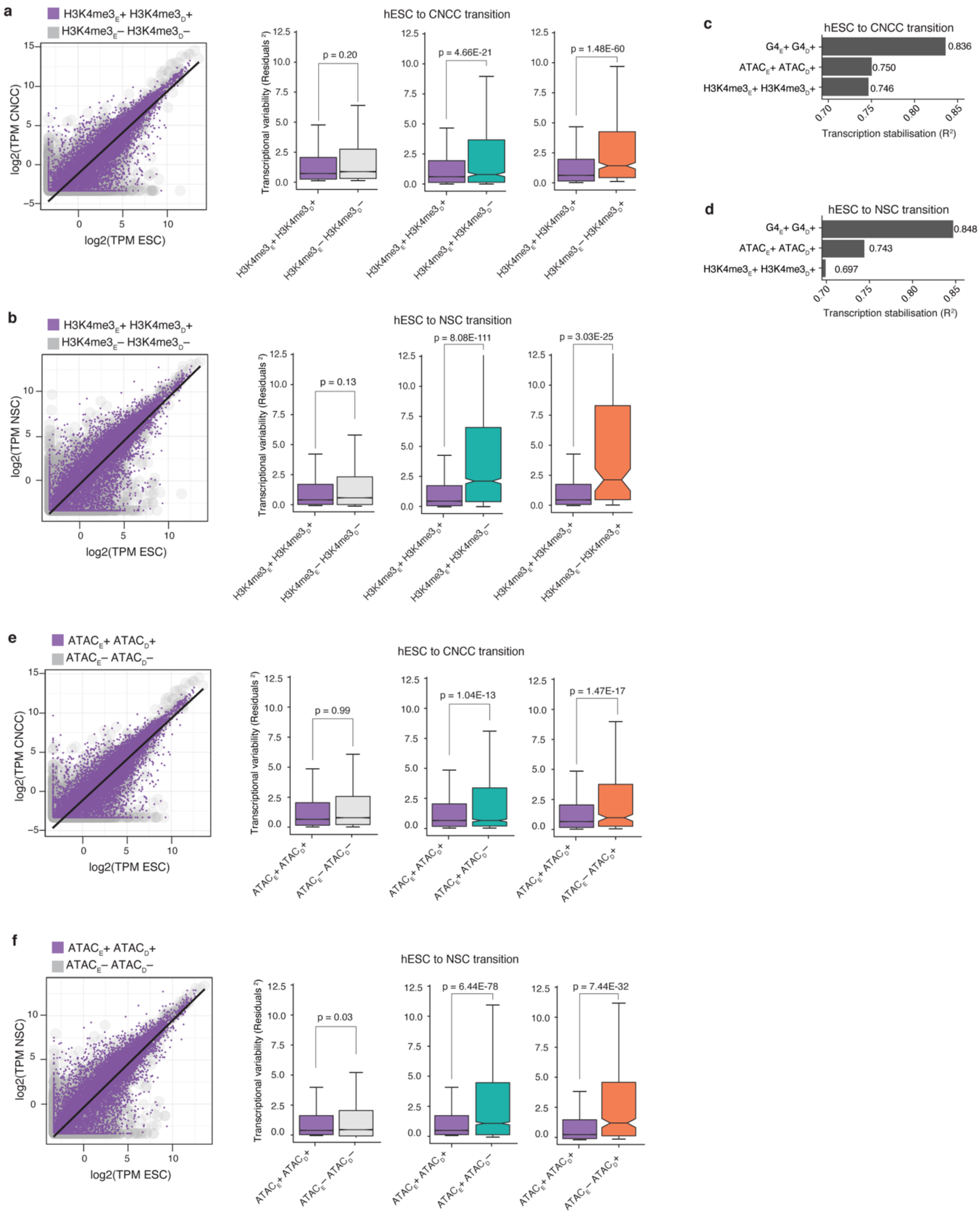
a
H3K4me3E+ H3K4me3D+
H3K4me3E– H3K4me3D–
log2(TPM CNCC)
log2(TPM ESC)
hESC to CNCC transition
Transcriptional variability (Residuals 2)
p = 0.20
p = 4.66E-21
p = 1.48E-60
H3K4me3E+ H3K4me3D+
H3K4me3E– H3K4me3D–
H3K4me3E+ H3K4me3D–
H3K4me3E– H3K4me3D+
b
H3K4me3E+ H3K4me3D+
H3K4me3E– H3K4me3D–
log2(TPM NSC)
log2(TPM ESC)
hESC to NSC transition
Transcriptional variability (Residuals 2)
p = 0.13
p = 8.08E-111
p = 3.03E-25
c
hESC to CNCC transition
G4E+ G4D+
ATACE+ ATACD+
H3K4me3E+ H3K4me3D+
0.836
0.750
0.746
Transcription stabilisation (R2)
d
hESC to NSC transition
G4E+ G4D+
ATACE+ ATACD+
H3K4me3E+ H3K4me3D+
0.848
0.743
0.697
Transcription stabilisation (R2)
e
ATACE+ ATACD+
ATACE– ATACD–
log2(TPM CNCC)
log2(TPM ESC)
hESC to CNCC transition
Transcriptional variability (Residuals 2)
p = 0.99
p = 1.04E-13
p = 1.47E-17
ATACE+ ATACD+
ATACE– ATACD–
ATACE+ ATACD–
ATACE– ATACD+
f
ATACE+ ATACD+
ATACE– ATACD–
log2(TPM NSC)
log2(TPM ESC)
hESC to NSC transition
Transcriptional variability (Residuals 2)
p = 0.03
p = 6.44E-78
p = 7.44E-32

Supplementary Figure 13: Maintenance of G4s during differentiation results in greater transcriptional stabilisation compared to H3K4me3 or open chromatin. a, Scatter plot (left) of the log2 median transcripts per million (TPM) for hESCs vs CNCCs with fitted weighted linear regression model (black line). $H3K4me3_E$; H3K4me3 promoter status in hESC and $H3K4me3_D$; H3K4me3 promoter status in daughter cells. Box plots (right) of the calculated residuals of $H3K4me3_E$+ $H3K4me3_D$+ and $H3K4me3_E$+ $H3K4me3_D$–, $H3K4me3_E$– $H3K4me3_D$+ or $H3K4me3_E$– $H3K4me3_D$–. Data are presented as median (centre) and interquartile range (box; the lower and upper bounds of the box represent the 25th and 75th percentiles, respectively). Whiskers represent ± 1.5x interquartile range. N = 5 and 3 biologically independent samples for hESCs and CNCCs, respectively. Number of genes per group: $H3K4me3_E$+ $H3K4me3_D$+ (15100) and $H3K4me3_E$– $H3K4me3_D$– (11619), $H3K4me3_E$+ $H3K4me3_D$– (1059) or $H3K4me3_E$– $H3K4me3_D$+ (506). One-tailed F- test for variances. **b,** Same analysis as described in (a) for hESC vs NSCs. N = 5 and 4 biologically independent samples for hESCs and NSCs, respectively. Number of genes per group: $H3K4me3_E$+ $H3K4me3_D$+ (14459) and $H3K4me3_E$– $H3K4me3_D$– (13036), $H3K4me3_E$+ $H3K4me3_D$– (1962) or $H3K4me3_E$– $H3K4me3_D$+ (182). **c-d,** Bar chart comparing transcriptional stabilisation, as determined by the coefficient of determination and goodness of fit, R^2, for genes which either maintain G4 ($G4_E$+ $G4_D$+), chromatin accessibility (defined by ATAC-seq, $ATAC_E$+ $ATAC_D$+) or H3K4me3 status ($H3K4me3_E$+ $H3K4me3_D$+) of their promoter upon (c) hESC to NSC or (d) hESC to CNCC differentiation. **e**, Scatter plot (left) of the log2 median transcripts per million (TPM) for hESCs vs CNCCs with fitted weighted linear regression model (black line). $ATAC_E$; ATAC promoter status in hESC and $ATAC_D$; ATAC promoter status in daughter cells. Box and whisker plots (right) of the calculated residuals for ATAC promoter classifications. Data are presented as median (centre) and interquartile range (box; the lower and upper bounds of the box represent the 25th and 75th percentiles, respectively). Whiskers represent ± 1.5x interquartile range. N = 5 and 3 biologically independent samples for hESCs and CNCCs, respectively. Number of genes per group: $ATAC_E$+ $ATAC_D$+ (16010) and $ATAC_E$– $ATAC_D$– (9869), $ATAC_E$+ $ATAC_D$– (2034) or $ATAC_E$– $ATAC_D$+ (371). One-tailed F- test for variances. **f,** Same analysis as described in (e) for hESC vs NSCs. N = 5 and 4 biologically independent samples for hESCs and NSCs, respectively. Number of genes per group: $ATAC_E$+ $ATAC_D$+ (15407) and $ATAC_E$– $ATAC_D$– (10856), $ATAC_E$+ $ATAC_D$– (3015) or $ATAC_E$– $ATAC_D$+ (361). One-tailed F- test for variances. See Supplementary Fig. 11-12 for full set of scatter plots used to calculate residuals.

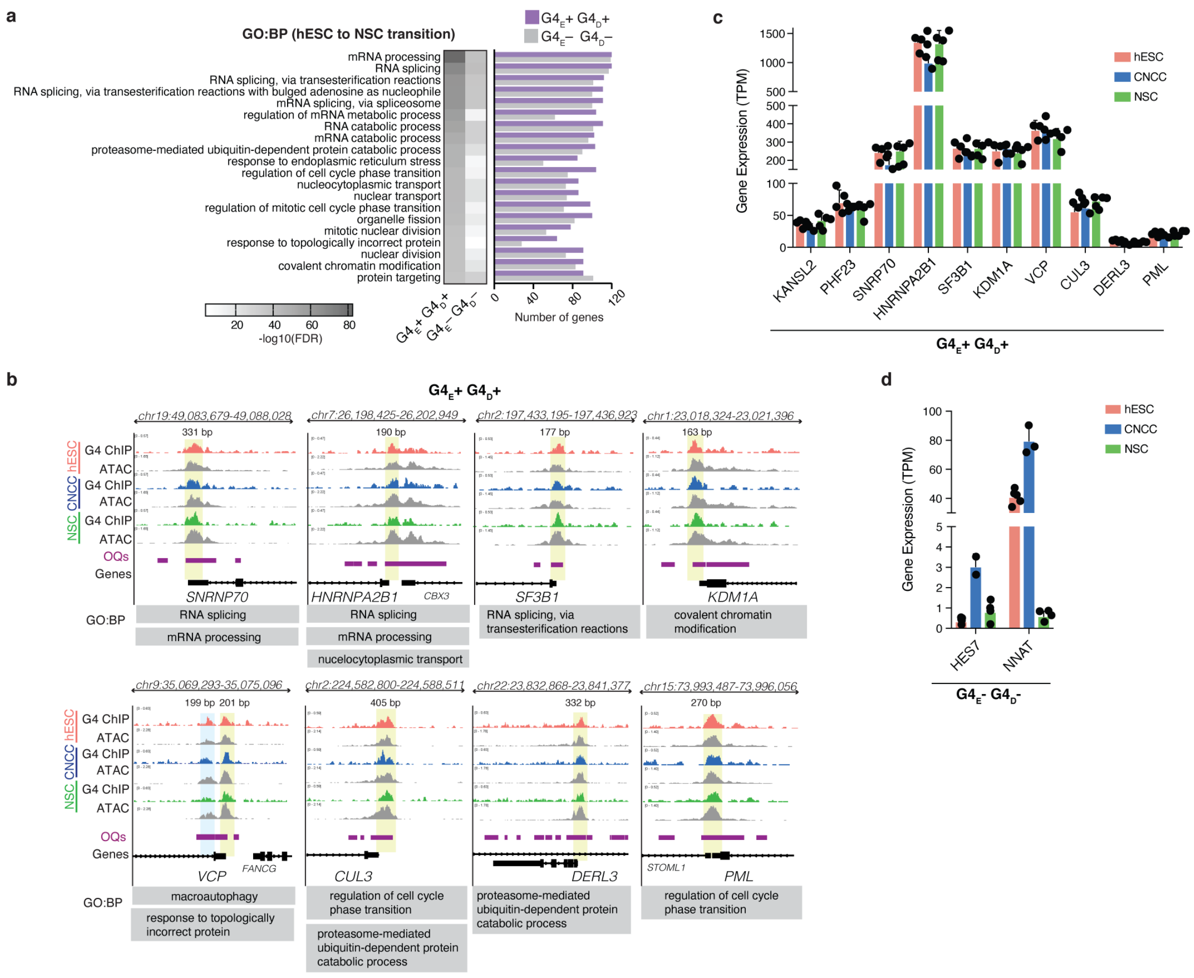

Supplementary Figure 14: Gene enrichment analysis for genes with a promoter G4 in both hESCs and daughter cells. a, Top 20 significant (FDR <0.05) biological processes obtained from Gene Ontology enrichment analysis (GO:BP) performed with g:Profiler[8] for genes that are not differentially expressed between hESCs and NSCs and maintain ($G4_E$+ $G4_D$+) a G4 (n = 2,696 genes) or never ($G4_E$– $G4_D$–) had a G4 (n = 4,073 genes) in their promoter. Number of genes intersecting GO term shown in adjacent bar plot. **b,** Genome browser view for representative gene promoters demonstrating $G4_E$+ $G4_D$+ promoter status after hESC differentiation into CNCCs or NSCs. Yellow shading highlights overlap of G4s, open chromatin sites (ATAC) and G4s that form *in vitro* (observed quadruplex sequences, OQs)[5]. **c-d,** Gene expression levels (transcript per million, TPM) for genes (as shown in b and Fig 4.e) which (c) maintain a G4 ($G4_E$+ $G4_D$+) or (d) never had a G4 in their promoter. Mean ± SD, N = 5, 3 and 4 biologically independent samples for hESCs, CNCCs and NSCs, respectively.

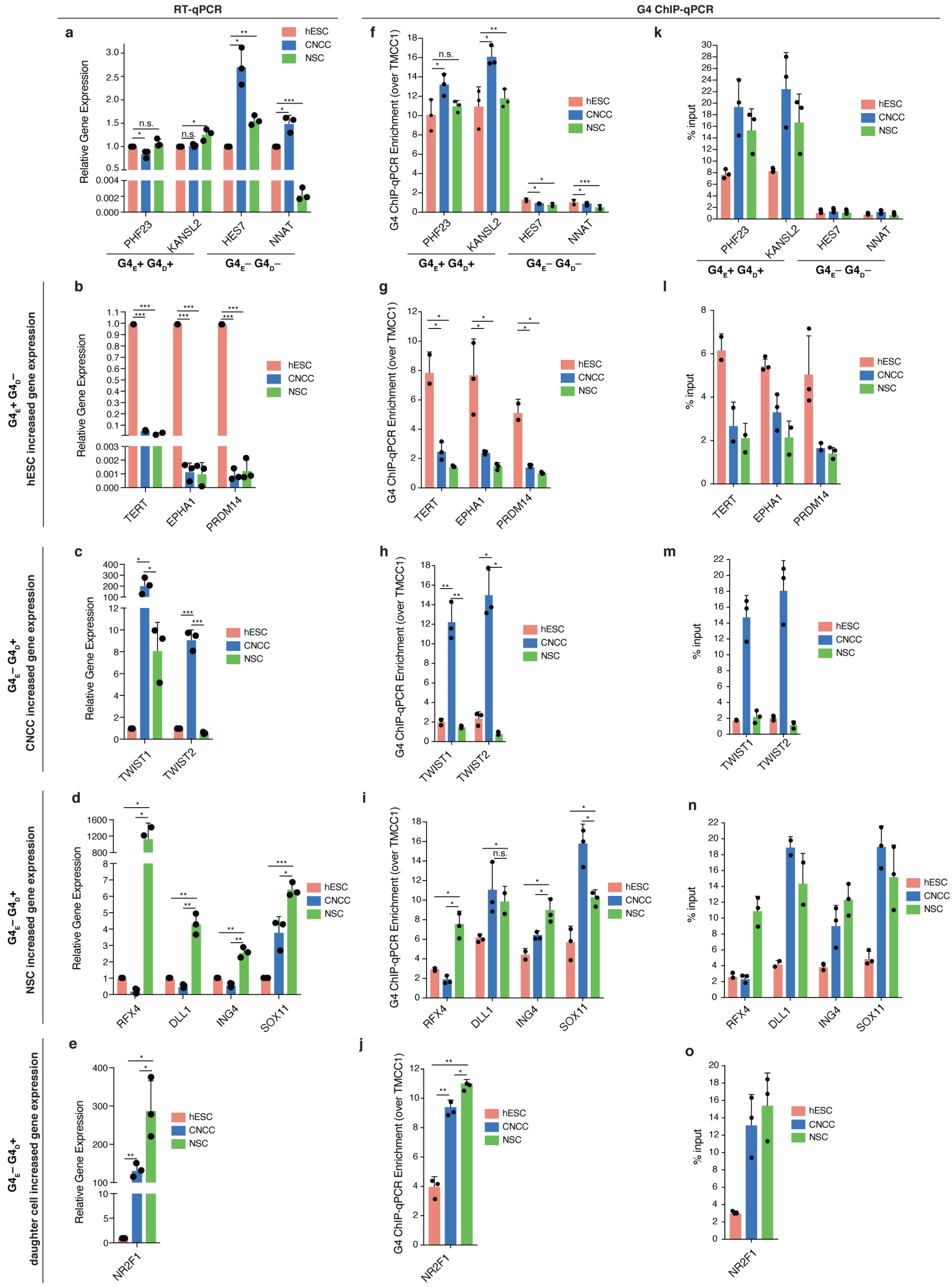
RT-qPCR
G4 ChIP-qPCR
a
f
k
b
g
l
c
h
m
d
i
n
e
j
o
hESC
CNCC
NSC
Relative Gene Expression
G4 ChIP-qPCR Enrichment (over TMCC1)
% input
PHF23
KANSL2
HES7
NNAT
G4E+ G4D+
G4E– G4D–
TERT
EPHA1
PRDM14
TWIST1
TWIST2
RFX4
DLL1
ING4
SOX11
NR2F1
n.s.
G4E+ G4D–
hESC increased gene expression
G4E– G4D+
CNCC increased gene expression
G4E– G4D+
NSC increased gene expression
G4E– G4D+
daughter cell increased gene expression

Supplementary Figure 15: RT-qPCR and G4-ChIP qPCR validation of selected genes uncovered by gene enrichment analyses. a-e, Relative gene expression levels (normalised to GAPDH and relative to hESCs) and **f-o,** G4 ChIP-qPCR of a selection of genes shown in Fig 4e; Fig 5 b,d,f; Supplementary Fig. 15. (f-j) The G4-negative *TMCC1* regulatory region[9] was used as internal reference to normalise G4-ChIP qPCR promoter enrichments. Mean ± SD, N = 3 biologically independent samples (see methods). Unpaired t-test, two-sided: *:p <0.05; **:p<0.001, ***: p <0.0001. See Source Data for exact p-values and Supplementary Data 4 for PCR primer sequences.

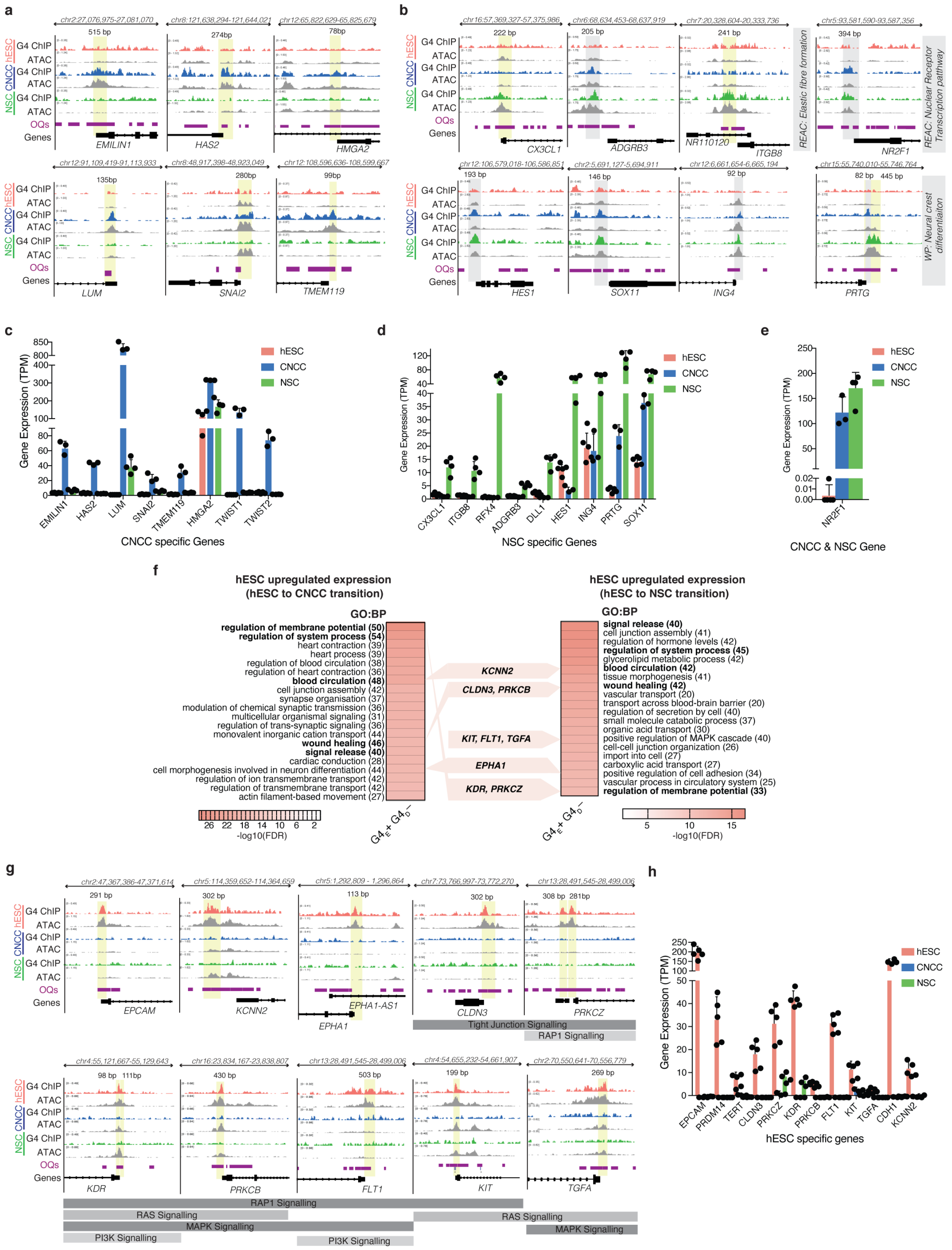

a
b
c
d
e
f
g
h
hESC
CNCC
NSC
G4 ChIP
ATAC
OQs
Genes
EMILIN1
HAS2
HMGA2
LUM
SNAI2
TMEM119
CX3CL1
ADGRB3
NR110120
ITGB8
NR2F1
HES1
SOX11
ING4
PRTG
REAC: Elastic fibre formation
REAC: Nuclear Receptor Transcription pathway
WP: Neural crest differentiaiton
Gene Expression (TPM)
CNCC specific Genes
NSC specific Genes
CNCC & NSC Gene
hESC upregulated expression
(hESC to CNCC transition)
(hESC to NSC transition)
GO:BP
regulation of membrane potential (50)
regulation of system process (54)
heart contraction (39)
heart process (39)
regulation of blood circulation (38)
regulation of heart contraction (36)
blood circulation (48)
cell junction assembly (42)
synapse organisation (37)
modulation of chemical synaptic transmission (36)
multicellular organismal signaling (31)
regulation of trans-synaptic signaling (36)
monovalent inorganic cation transport (44)
wound healing (46)
signal release (40)
cardiac conduction (28)
cell morphogenesis involved in neuron differentiation (44)
regulation of ion transmembrane transport (42)
regulation of transmembrane transport (42)
actin filament-based movement (27)
signal release (40)
cell junction assembly (41)
regulation of hormone levels (42)
regulation of system process (45)
glycerolipid metabolic process (42)
blood circulation (42)
tissue morphogenesis (41)
wound healing (42)
vascular transport (20)
transport across blood-brain barrier (20)
regulation of secretion by cell (40)
small molecule catabolic process (37)
organic acid transport (30)
positive regulation of MAPK cascade (40)
cell-cell junction organization (26)
import into cell (27)
carboxylic acid transport (27)
positive regulation of cell adhesion (34)
vascular process in circulatory system (25)
regulation of membrane potential (33)
KCNN2
CLDN3, PRKCB
KIT, FLT1, TGFA
EPHA1
KDR, PRKCZ
-log10(FDR)
EPCAM
KCNN2
EPHA1-AS1
EPHA1
CLDN3
PRKCZ
KDR
PRKCB
FLT1
KIT
TGFA
Tight Junction Signalling
RAP1 Signalling
RAS Signalling
MAPK Signalling
PI3K Signalling
hESC specific genes

Supplementary Figure 16: Genome browser views for selected genes uncovered by gene enrichment analysis that maintain a G4 promoter during differentiation of hESCs to daughters. a-b, Genome browser view for representative gene promoters demonstrating $G4_E$– $G4_D$+ promoter status after hESC differentiation into (a) CNCCs or (b) NSCs from enriched GO: BP terms (see Fig. 5 a and c). Yellow shading highlights location of cell specific G4 across open chromatin sites (ATAC) and G4s that can form *in vitro* (observed quadruplex sequences, OQs)[5]. Grey shading highlights G4 common to both CNCCs and NSCs but absent in hESCs. **c-e,** Gene expression levels (transcript per million, TPM) for genes shown in a, b and Fig 5 a and d. Mean ± SD, N = 5, 3 and 4 biologically independent samples for hESCs, CNCCs and NSCs, respectively (see methods). **f,** Top 20 significant (FDR< 0.05) GO: BP terms for genes which have increased expression in hESC compared to CNCC (n = 1,128 genes) or NSC (n = 1,175 genes) which lose a promoter G4 ($G4_E$+ $G4_D$–). Number of genes in intersection for each term shown in brackets. See Supplementary Data 2 and 3 for full list of terms. **g,** Genome browser view for representative gene promoters demonstrating $G4_E$+ $G4_D$+ promoter status after hESC differentiation into CNCCs or NSCs. Yellow box highlights hESC specific G4s and their overlap with open chromatin sites (ATAC) and G4s that can form *in vitro* (OQs)[5]. **h,** Gene expression levels (transcript per million, TPM) for genes shown in g and Fig 5f which lose a G4 ($G4_E$+ $G4_D$–) G4 in their promoter. Mean ± SD, N = 5, 3 and 4 biologically independent samples for hESCs, CNCCs and NSCs, respectively (see methods).

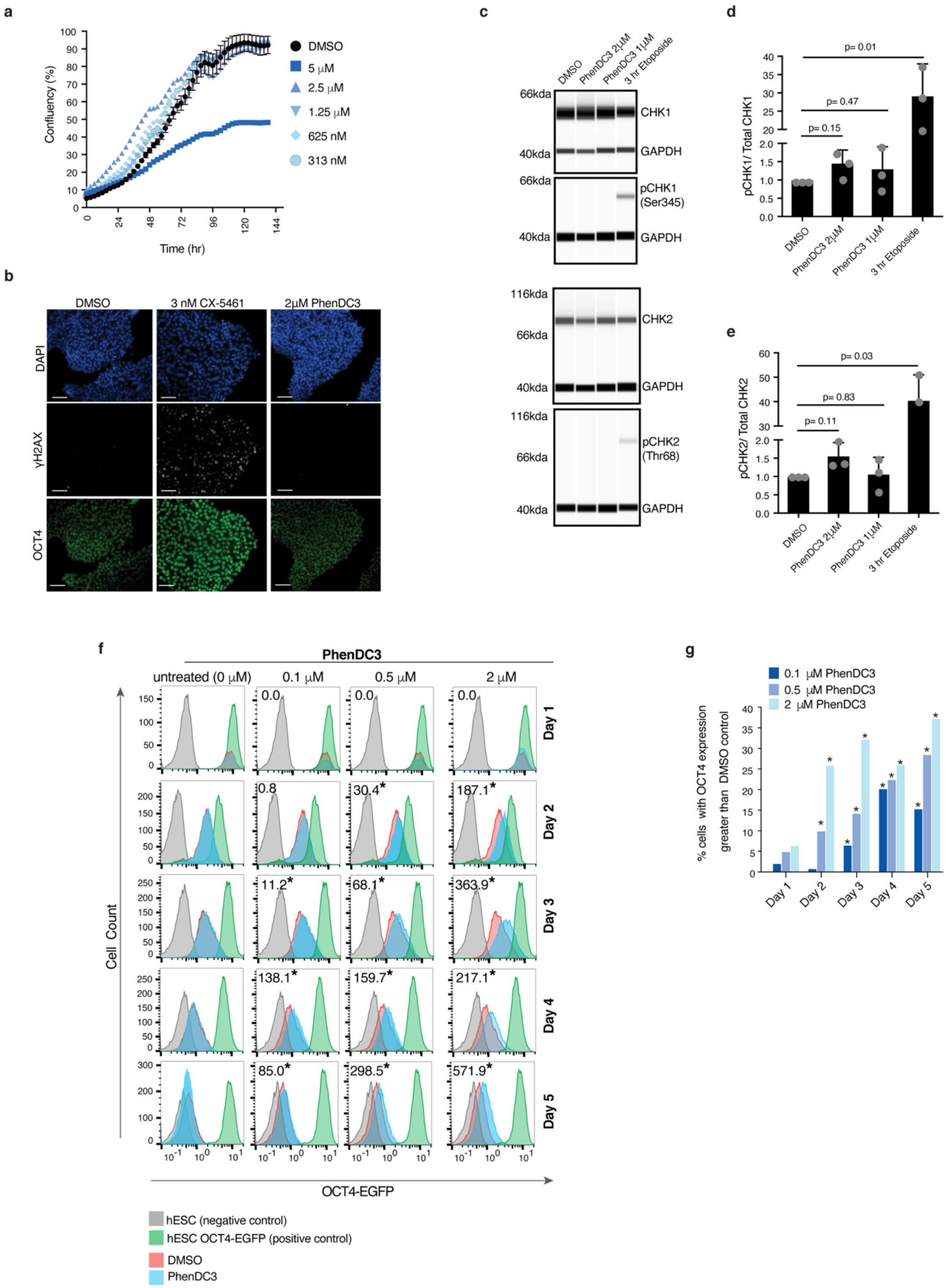
a
Confluency (%)
Time (hr)
DMSO
5 µM
2.5 µM
1.25 µM
625 nM
313 nM
b
DMSO
3 nM CX-5461
2µM PhenDC3
DAPI
γH2AX
OCT4
c
DMSO
PhenDC3 2µM
PhenDC3 1µM
3 hr Etoposide
66kda
40kda
116kda
CHK1
GAPDH
pCHK1
(Ser345)
CHK2
pCHK2
(Thr68)
d
pCHK1/ Total CHK1
p= 0.01
p= 0.47
p= 0.15
e
pCHK2/ Total CHK2
p= 0.03
p= 0.83
p= 0.11
f
PhenDC3
untreated (0 µM)
0.1 µM
0.5 µM
2 µM
Day 1
Day 2
Day 3
Day 4
Day 5
Cell Count
OCT4-EGFP
0.0
0.8
30.4*
187.1*
11.2*
68.1*
363.9*
138.1*
159.7*
217.1*
85.0*
298.5*
571.9*
hESC (negative control)
hESC OCT4-EGFP (positive control)
DMSO
PhenDC3
g
0.1 µM PhenDC3
0.5 µM PhenDC3
2 µM PhenDC3
% cells with OCT4 expression greater than DMSO control

Supplementary Figure 17: Differentiation delay with PhenDC3 treatment is not due to growth arrest or induction of DNA damage. a, Growth curves for OCT4-EGFP hESCs grown in mTESR1 media on Matrigel over 144 hours in the presence of 0.02% DMSO (black) or the G4-specific ligand PhenDC3 at indicated concentrations. Error bars represent the Mean ± SD of measurements from 3 independent wells. **b,** Representative IF images of OCT4-EGFP hESCs stained with antibodies against γH2AX and OCT4 after 5 day treatment with 0.02% DMSO as negative control, the G4 ligand CX-5461 known to induce DNA damage[10] as positive control or PhenDC3. Scale Bar = 100 µm. **c,** hESCs were differentiated to CNCCs in the presence of PhenDC3 (1 µM and 2 µM) or 0.2% DMSO. On day 5, cell lysates were probed with antibodies against CHK1, phosphorylated CHK1(Ser354), CHK2, phosphorylated CHK2 (Thr68) and GAPDH control by western blotting. **d-e**, Normalised protein levels for d) pCHK1 and e) pCHK2. N = 3 independent biological replicates (Mean ± SD), Welch's t-test, two-tailed. hESCs treated with 100 nM etoposide for 3 hours was used as a positive control. **f-g,** OCT4-EGFP expression levels over 5-day CNCC differentiation obtained from live cell flow cytometry. (f) FACs histogram plots and (g) calculated proportion of cells with higher levels of OCT4-EGFP expression compared to DMSO controls. χ^2 values, calculated using the Chi squared T(x) test in 'compare populations' using FlowJo software, for each PhenDC3 vs DMSO comparison are shown in top-left of each FACs histogram. *: comparisons are statistically different ($p < 0.01$, T(x) metric) compared to untreated (0 µM PhenDC3 and no DMSO) vs 0.02% DMSO (see Methods). N = 10,000 cells analysed per day (apart from day 1 where the whole sample was analysed (5,000 cells)). N = one independent biological replicate. See Source Data.

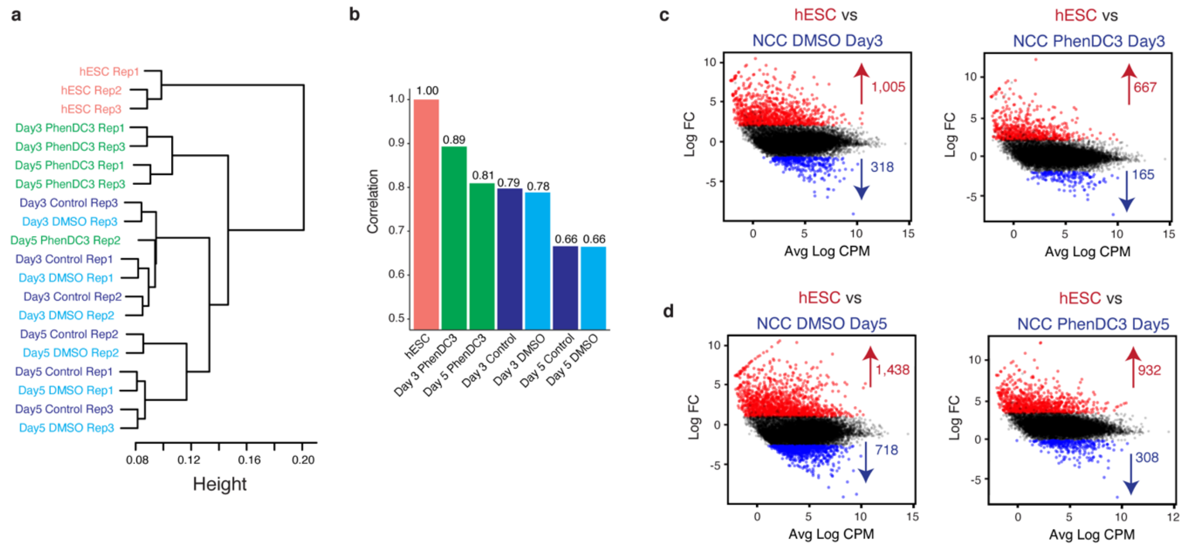

Supplementary Figure 18: PhenDC3 treatment results in differentiation delay. RNA-seq was performed at day 3 and day 5 after induction of the 5-day CNCC differentiation protocol. hESCs grown in mTESR1 were used as the pluripotency control. **a,** Hierarchical clustering of counts per million (CPM) generated from 3 biological replicates (i.e. Rep) at day 3 and day 5 for hESCs treated with NCC induction media only (Control) or with NCC induction media and DMSO or 2uM PhenDC3. Height calculated as 1-euclidean distance. **b,** Spearman correlation of expression levels (average counts per million) for stem cell maintenance genes (GO:0019827). **c-d** MA plots showing differential expressed genes (Log2FC >2, FDR <0.05) for the comparison between hESC and DMSO treated (left) and hESC and 2 μM PhenDC3 treated after NCC induction (right) at (c) day 3 and (d) day 5.

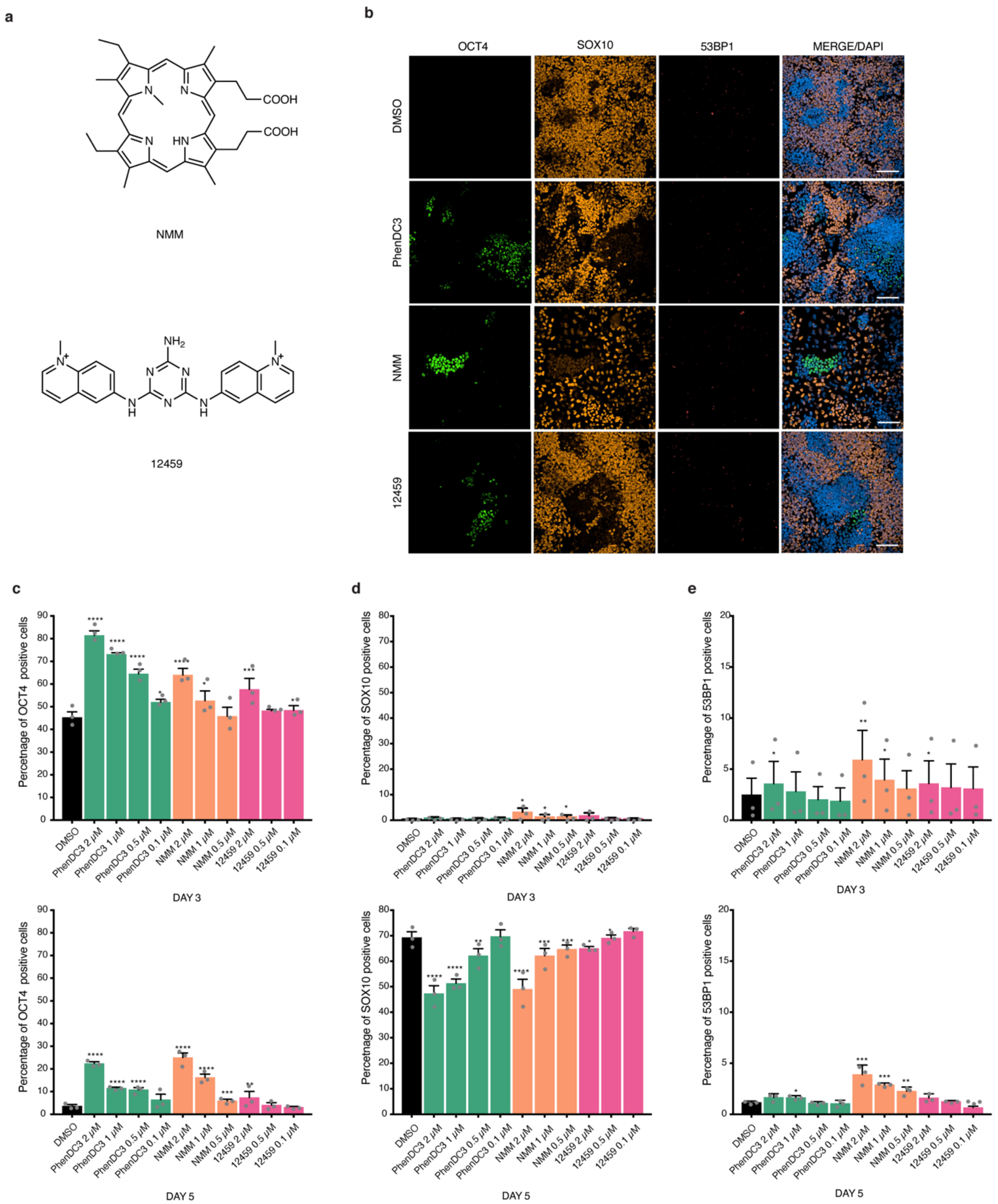
a
COOH
COOH
NMM
NH_2
12459
b
OCT4
SOX10
53BP1
MERGE/DAPI
DMSO
PhenDC3
NMM
12459
c
Percetnage of OCT4 positive cells
DAY 3
DAY 5
d
Percetnage of SOX10 positive cells
DAY 3
DAY 5
e
Percetnage of 53BP1 positive cells
DAY 3
DAY 5
DMSO
PhenDC3 2 µM
PhenDC3 1 µM
PhenDC3 0.5 µM
PhenDC3 0.1 µM
NMM 2 µM
NMM 1 µM
NMM 0.5 µM
12459 2 µM
12459 0.5 µM
12459 0.1 µM

Supplementary Figure 19: Treatment with further G4-specific small molecules results in hESC differentiation delay. hESCs were differentiated into CNCCs as described in Figure 6. Samples were taken at the day 3 and day 5 to determine the percentage of cells positive for the pluripotency marker OCT4 or SOX10 (lineage marker for CNCC) by immunofluorescence (IF). **a**, Chemical structures of G4-specific ligands *N*-methyl mesoporphyrin IX (NMM)[11,12] and 12459[13]. **b**, Representative confocal IF images of differentiating hESCs treated with either 0.02% DMSO, 2 μM PhenDC3, 2 μM NMM or 2 μM 12459. SOX10 = red, OCT4 = green and DAPI (nuclear stain) = blue. Scale bar = 100 μm. **c-d**, Proportion of (c) OCT4, (d) SOX10 and (e) 53BP1 (DNA damage marker[14]) positive cells determined in IF studies. n = 7,000 to 75,000 cells obtained from 52 fields of view per sample. Mean ± SEM shown for 3 independent biological replicates *: $p < 0.05$, **: $p < 1E-10$, ***: $p < 1E-50$, ****: $p < 1E-100$; one-sided Pearson's χ^2 test for proportions (see Source Data for exact p-values).

Supplementary Discussion

hESC cell cycle changes do not account for high G4 abundance

We considered whether hESCs have more G4s due to a higher proportion of asynchronously dividing cells in S phase[15] as the increased transient occurrence of single-strand DNA during replication could favour G4 formation[16]. To rule this out, cell cycle analysis of asynchronous hESCs, CNCCs and NSCs was performed using flow cytometry. As expected, there is an increased percentage of cells in G1 in CNCCs and NSCs compared to hESC, due to lengthening of the G1 phase which accompanies differentiation[17,18] (Supplementary Fig. 3c-e). Notably, the fraction of CNCCs in S phase (55.4% ± 2.7%) was not significantly lower than hESCs (54.3% ± 1.8%) ($p = 0.800$) thus the number of cells in S-phase alone does not account for the high number of G4s in hESCs.

Expression level differences across promoter classes

Compared to genes carrying promoters marked by H3K4me3, promoters positive for both active H3K4me3 and repressive H3K27me3 marks (bivalent) are generally either transcriptional silent or lowly expressed[19]. To determine whether G4 presence in a promoter is associated with their transcriptional output, the 4 histone promoter signatures (H3K4me3, bivalent, H3K27me3 and unmarked) were stratified by the presence or absence of a G4 (G4+, G4–, respectively). In all 3 stem cell types, genes with H3K4me3 or bivalent promoters that contained a G4 showed significantly ($p \leq 6.0E-09$) increased transcription compared to those without at G4. However bivalent G4-positive promoters had significantly ($p \leq 6.9E-5$) lower expression than H3K4me3 genes (Fig. 3h, Supplementary Fig. 9a-b). This demonstrates that the presence of a G4 in a promoter leads to on average higher expression levels, supporting our previous observations in cancer[20]. However, for bivalent promoters, promoter histone classification appears to determine transcriptional levels relative to G4 presence.

To assess if the G4 landscape alone directly recapitulates transcriptional levels, we performed a k-medoid clustering of the 11,876 genes (total 58,381 gene set filtered for genes with median TPM >1) considering normalised expression (median TPM) levels and promoter G4 signal (median from replicates) for each gene across the three stem cell types (Supplementary Fig. 9d-f). This clustering analysis revealed that G4 levels do not directly account for expression levels, thus G4s are not sufficient to recapitulate overall expression patterns. Furthermore, this integrative analysis revealed 6 district trends and that G4 promoters can be associated with all ranges of expression levels.

Supplementary References

1. Prescott, S. L. *et al.* Enhancer Divergence and cis-Regulatory Evolution in the Human and Chimp Neural Crest. *Cell* **163**, 68–83 (2015).
2. Xie, W. *et al.* Epigenomic analysis of multilineage differentiation of human embryonic stem cells. *Cell* **153**, 1134–1148 (2013).
3. Machanick, P. & Bailey, T. L. MEME-ChIP: Motif analysis of large DNA datasets. *Bioinformatics* (2011) doi:10.1093/bioinformatics/btr189.
4. Grant, C. E., Bailey, T. L. & Noble, W. S. FIMO: Scanning for occurrences of a given motif. *Bioinformatics* (2011) doi:10.1093/bioinformatics/btr064.
5. Marsico, G. *et al.* Whole genome experimental maps of DNA G-quadruplexes in multiple species. *Nucleic Acids Res.* **47**, 3862–3874 (2019).
6. Biffi, G., Tannahill, D., McCafferty, J. & Balasubramanian, S. Quantitative visualization of DNA G-quadruplex structures in human cells. *Nat. Chem.* **5**, 182–186

(2013).
7. Lee, S. M. *et al.* Intragenic CpG islands play important roles in bivalent chromatin assembly of developmental genes. *Proc. Natl. Acad. Sci. U. S. A.* **114**, E1885–E1894 (2017).
8. Raudvere, U. *et al.* G:Profiler: A web server for functional enrichment analysis and conversions of gene lists (2019 update). *Nucleic Acids Res.* (2019) doi:10.1093/nar/gkz369.
9. Hänsel-Hertsch, R., Spiegel, J., Marsico, G., Tannahill, D. & Balasubramanian, S. Genome-wide mapping of endogenous G-quadruplex DNA structures by chromatin immunoprecipitation and high-throughput sequencing. *Nat. Protoc.* **13**, 551–564 (2018).
10. Xu, H. *et al.* CX-5461 is a DNA G-quadruplex stabilizer with selective lethality in BRCA1/2 deficient tumours. *Nat. Commun.* **8**, 1–18 (2017).
11. Yingfu Li, C. Ronald Geyer, and & Sen*, D. Recognition of Anionic Porphyrins by DNA Aptamers†. *Biochemistry* **35**, 6911–6922 (1996).
12. Nicoludis, J. M., Barrett, S. P., Mergny, J.-L. & Yatsunyk, L. A. Interaction of human telomeric DNA with N- methyl mesoporphyrin IX. *Nucleic Acids Res.* **40**, 5432–5447 (2012).
13. Riou, J. F. *et al.* Cell senescence and telomere shortening induced by a new series of specific G-quadruplex DNA ligands. *Proc. Natl. Acad. Sci.* **99**, 2672–2677 (2002).
14. Rappold, I., Iwabuchi, K., Date, T. & Chen, J. Tumor Suppressor P53 Binding Protein 1 (53bp1) Is Involved in DNA Damage–Signaling Pathways. *J. Cell Biol.* **153**, 613 (2001).
15. Becker, K. A. *et al.* Self-renewal of human embryonic stem cells is supported by a shortened G1 cell cycle phase. *J. Cell. Physiol.* **209**, 883–893 (2006).
16. Técher, H., Koundrioukoff, S., Nicolas, A. & Debatisse, M. The impact of replication stress on replication dynamics and DNA damage in vertebrate cells. *Nature Reviews Genetics* vol. 18 535–550 (2017).
17. Calegari, F. & Huttner, W. B. An inhibition of cyclin-dependent kinases that lengthens, but does not arrest, neuroepithelial cell cycle induces premature neurogenesis. *J. Cell Sci.* **116**, 4947–4955 (2003).
18. Borghese, L. *et al.* Inhibition of Notch Signaling in Human Embryonic Stem Cell-Derived Neural Stem Cells Delays G1/S Phase Transition and Accelerates Neuronal Differentiation In Vitro and In Vivo. *Stem Cells* **28**, 955–964 (2010).
19. Bernstein, B. E. *et al.* A Bivalent Chromatin Structure Marks Key Developmental Genes in Embryonic Stem Cells. *Cell* **125**, 315–326 (2006).
20. Hänsel-Hertsch, R. *et al.* G-quadruplex structures mark human regulatory chromatin. *Nat. Genet.* **48**, 1267–1272 (2016).
