# Supplementary material for: G-quadruplex DNA structures in human stem cells and differentiation
Source: Nat Commun. 2022 Jan 10;13:142. doi: 10.1038/s41467-021-27719-1 (PMC8748810; doi:10.1038/s41467-021-27719-1)
Supplement: Supplementary file 3 — Description of Additional Supplementary Files [file 41467_2021_27719_MOESM3_ESM.pdf]

Description for additional supplementary data files

Supplementary Data 1: Enriched Gene Ontology Biological Processes, KEGG, Reactome and Wikipathway terms obtained for g:Profiler analysis for Supplementary Fig. 8b and g. See README tab for further details. Gene set enrichment analysis was performed using a rightsided (enrichment) hyper-geometric test with a Benjamini-Hochberg adjustment of the resulting p-value.

Supplementary Data 2: Enriched Gene Ontology Biological Processes, KEGG, Reactome and Wikipathway terms obtained for g:Profiler analysis for hESC to CNCC transition in Fig. 4d, Fig.5a and e and Supplementary Fig. 16f. See README tab for further details. Gene set enrichment analysis was performed using a right-sided (enrichment) hyper-geometric test with a Benjamini-Hochberg adjustment of the resulting p-value. 31

Supplementary Data 3: Enriched Gene Ontology Biological Processes, KEGG, Reactome and Wikipathway terms obtained for g:Profiler analysis hESC to NSC transition in Fig. 5c, Supplementary Fig. 14a and Supplementary Fig. 16f. See README tab for further details. Gene set enrichment analysis was performed using a right-sided (enrichment) hypergeometric test with a Benjamini-Hochberg adjustment of the resulting p-value.

Supplementary Data 4: Primer sequences used for RT-qPCR and G4-ChIP-qPCR in Supplementary Fig. 15.
