# Supplementary material for: G-quadruplex DNA structures in human stem cells and differentiation
Source: Nat Commun. 2022 Jan 10;13:142. doi: 10.1038/s41467-021-27719-1 (PMC8748810; doi:10.1038/s41467-021-27719-1)
Supplement: Supplementary file 8 — Reporting Summary [file 41467_2021_27719_MOESM8_ESM.pdf]

nature research

Corresponding author(s): Shankar Balasubramanian

Last updated by author(s): Nov 16, 2021

Reporting Summary

Nature Research wishes to improve the reproducibility of the work that we publish. This form provides structure for consistency and transparency in reporting. For further information on Nature Research policies, see our Editorial Policies and the Editorial Policy Checklist.

Statistics

For all statistical analyses, confirm that the following items are present in the figure legend, table legend, main text, or Methods section.

n/a	Confirmed	
☐	☒	The exact sample size (*n*) for each experimental group/condition, given as a discrete number and unit of measurement
☐	☒	A statement on whether measurements were taken from distinct samples or whether the same sample was measured repeatedly
☐	☒	The statistical test(s) used AND whether they are one- or two-sided<br>*Only common tests should be described solely by name; describe more complex techniques in the Methods section.*
☐	☒	A description of all covariates tested
☐	☒	A description of any assumptions or corrections, such as tests of normality and adjustment for multiple comparisons
☐	☒	A full description of the statistical parameters including central tendency (e.g. means) or other basic estimates (e.g. regression coefficient) AND variation (e.g. standard deviation) or associated estimates of uncertainty (e.g. confidence intervals)
☐	☒	For null hypothesis testing, the test statistic (e.g. *F*, *t*, *r*) with confidence intervals, effect sizes, degrees of freedom and *P* value noted<br>*Give P values as exact values whenever suitable.*
☒	☐	For Bayesian analysis, information on the choice of priors and Markov chain Monte Carlo settings
☒	☐	For hierarchical and complex designs, identification of the appropriate level for tests and full reporting of outcomes
☒	☐	Estimates of effect sizes (e.g. Cohen's *d*, Pearson's *r*), indicating how they were calculated

Our web collection on statistics for biologists contains articles on many of the points above.

Software and code

Policy information about availability of computer code

Data collection	ChIP-seq, ATAC-seq and RNA-seq data was collected on NextSeq500 and HiSeq4000. Immunofluorescence image collection was performed on Harmony High-Content Imaging and Analysis Software (PerkinElmer, ver: 4.9) and Leica Application Suite X (LAS X, Leica Microsystems). Flow cytometry data sets were collected through MACSQuantify Software (ver: 2.11.5) on a MACSQuant Analyzer (Miltenyi Biotec GmbH) and BD FACSDiva software ver: 2.0) on a FACs Aria Cell Sorter (BD Biosciences). qPCR experiments were collected using the Bio-Rad CFX manager Software (ver: 3.1). Western blots were performed on a Wes Protein Simple Western System. Live cell image collection was performed using IncuCyte ZOOM Live-Cell Analysis System (Sartorius). Further information is described in the methods.
Data analysis	ChIP-seq processing: After quality controls checks with fastQC (FasQC v0.11.7), fastq reads were trimmed from adapters using cutadapt (-m 10 -q 20 -O 3 -a CTGTCTCTTATACACATCT, ver: v0.11.7) and aligned to the human genome hg38 with bwa mem (ver: 0.7.17-r1188). Bam files were generated from alignment with samtools (ver: 1.11) view and duplicated reads were marked and removed using picard MarkDuplicates (picard-2.20.3). Total number of unique reads was quantified for each library with samtools view -c. Regions with local enrichments were obtained by calling peaks with macs2 (ver: 2.1.2) (--broad option) for each individual pull-down library paired to the corresponding input control. Consensus regions of each biological replicate was obtained as the regions observed in 2 out of the 3 technical replicates (multiIntersectBed, bedtools ver: v2.27.1). The final cell-type consensus was obtained by selecting regions reproducibly observed in at least 2 of the 3 biological replicates. Genome-wide reads per million (RPM) G4 signal was obtained by quantifying the read coverage across the genome and scaling it to a factor that reflected the individual library size (deeptools bamCoverage -scaleFactor, where factor = 1,000,000/ Lib_size, deeptools ver: 3.5.0). Differential G4-binding was carried out with edgeR (edgeR_3.26.8). Initially, library size and read coverage at a multi-cell G4 consensus (merging all three cells individual G4 consensus) regions were computed. Then, a generalized linear model (glmLRT, edgeR) with default parameters (negative binomial log-linear distribution of read counts) was used to assess regions with differential binding signal. Specifically, the differential binding analysis compared each cell to one of the other two in turn and regions with differential signal were identified as those with FDR <0.05.<br><br>ATAC-seq processing: After quality controls checks with fastQC (ver: 0.11.7), fastq reads were trimmed from adapters using cutatapt (cutadapt

-a AGATCGGAAGAGC -A AGATCGGAAGAG). Resulting reads were aligned to hg38 with bwa mem -M -t 12. Barn files were generated by using samtools view -Sb -F780 -q 10 -L (ver: 1.8). All libraries were sequenced twice and processed and aligned separately. Resulting alignments were merged and sorted. Duplicates were marked by picard MarkDuplicates (ver: 2.20.3, http://broadinstitute.github.io/picard) and removed. Fragment size distribution was estimated using uniquely mapped reads bams with picard CollectInsertSizeMetric. To assess the amount of mitochondria contamination, reads mapping to ChM were identified and counted directly from the alignment bam files. For each library, regions with local accessibility were identified by calling peaks with macs2 with default options and excluding chrM. For each cell type (hESCs, CNCCs and NSCs) peak regions observed in 2 out the 3 biological replicates were selected as the consensus regions using bedtools multiIntersectBed (ver: 2.27.1). The consensus for each cell type was then used for all subsequent analysis.

RNA-seq processing: After quality controls checks with fastQC (ver: 0.11.7), fastq reads were trimmed from adapters with cutadapt (cutadapt -q 20 -m 20 -a AGATCGGAAGAGC, ver: 1.16) and then aligned to hg38 by using rsem (rsem-calculate-expression --phred33-quals -p 40 --bowtie2, ver: 1.3.1) and files containing genes and isoform level expression estimates were produced. Genes level expression estimates files (genes.results) were used for the subsequent analysis. When technical replicates were present, TPM (transcript per million) from the expected counts from the gene levels estimate files(genes.results) were averaged. As a quality control, the similarity across technical replicates was assessed by computing correlation between TPM and Pearson correlation was R>0.95. Similarity across biological replicates was assessed by hierarchical clustering (experiments) paired to k-mean (promoters, with k=2) by using Heatmap from the complexHeatmap R package (ComplexHeatmap_2.0.0) (hierarchical clustering using Euclidean distance and average linkage).
The differential gene expression analysis between each pair of cells (hESCs versus CNCCs, hESCs versus NSCs and NSCs versus CNCCs) was performed with EdgeR using the expected_counts from the gene.results files generated during the alignment step. For each pairwise comparison, genes with differential expression (FDR <0.05 and abs(log2FC)>1) were called using the function glmQLFit taking into account the presence of multiple biological replicates in the experimental design matrix (5 for hESCs, 3 for CNCCs and 4 for NSCs). For each cell type expression levels have been summarized as the median TPM observed across all the biological replicates.

Analysis of transcriptional stabilisation: Transcriptional stabilisation and variability were explored in relation to the G4 promoter signatures (that capture how G4 regions are progressing from hESC (E) to each of the two daughter cells (D)) in two approaches:
Firstly, gene expression (TPM) levels were directly compared between hESCs and daughter cells. Only genes with TPM>0 in at least one of the two cells under investigation were considered. For each promoter group (G4E+G4D-, G4E+G4D+, G4E+G4D+, G4E-G4D-), we fitted a weighted linear regression to model the relationship between the two sets of expression levels. The weights used in the fitting are expression levels of hESCs that represent conceptually the reference starting condition. Residuals of the data from the regressed model were computed and used to quantifying the spread of the transcriptional variability. After each fitting step, the coefficient of "goodness of fit" R2 was computed and the F-test was used to assess if there were significant differences in transcriptional stability between pairs of promoter groups. A similar analysis was performed using our ATAC-seq data and published H3K4me3 ChIP-seq data. In each of the two cases, promoters were divided into 4 groups based on the presence/absence of the mark at promoters in hESCs/daughter cells. The ranking of R2 values was used to cross compare and determine which feature (G4, accessibility or H3K4me3) had a greater impact on stabilizing expression when the feature is transmitted from hESCs to daughter cells.
Secondly, transcriptional stabilisation was analysed using differential gene expression. For the 4 promoter classes, the proportion of genes belonging to each of the following groups was determined: proportion of genes differentially UP, proportion of genes differentially DOWN and proportion of genes not differentially expressed For each promoter class, we obtained 3 proportions and tested differences using proportion test (R function prop.test(), Pearson's chi-square test for proportions).

R-session information: R version 3.6.1 (2019-07-05) used for downstream genomic and transcriptomic computational analyses.

Functional analysis: Gene ontology, KEGG pathways, Reactome pathways and Wikipathways enrichment analysis was performed by using g:Profiler. Gene lists of interest were used as queries to g:Profiler web-server and enrichments were evaluated by a right-sided test based on the hyper-geometric distribution; statistical significance was expressed as corrected Benjamini-Hockbeng p-values. Term size was limited to the range 20-450 genes.

De-novo motif discovery: motif analysis of fasta sequences of the consensus genomic regions of interest was performed with MEME-ChIP (options: Enrichment mode Classic; Set of known motifs: Eukaryote DNA, Human and Mouse (HOCOMOCO v11 FULL), order-1Background, MEME Site Distribution: 0 or 1 occurrence, MEME motif count: 3 and MEME Motif width: 6-30 wide (inclusive). FIMO was used to evaluate the occurrence of selected top hit motives.

G4 fold-enrichments at sites of interests: fold enrichments at (epi-)genomic regions were computed by using the Genomic Association Tester (GAT, https://gat.readthedocs.io/en/latest/contents.html, 1000 randomisations) and the analysis was restricted to the human whitelist.

G4 immunofluorescence microscopy image analysis: Images were deconvolved using Huygens Professional Software (Scientific Volume Imaging BV). G4 and DAPI signal intensity of images was performed with ImageJ FIJI version 2.0 and and ICY (http://icy.bioimageanalysis.org) open software platforms. After identification of regions of interest (ROIs) based on DAPI channel using a Gaussian Blur (sigma=3), Huang dark automatic thresholding and Watershed function to separate touching objects. ROIS were used in ICY protocol to determine G4 signal density (sum G4 signal/ sum DAPI signal) per nuclei.

5-day neural crest differentiation experiment image analysis: was performed using Harmony High Content Analysis System (ver: 4.9, PerkinElmer). See methods "Immunofluorescence Image Analysis: 5-day neural crest differentiation experiment" for further details.

Cell cycle analysis: flow cytometry used to determine cell number in G1, G2/M and S from a population of 10,000 cells (MACSquant Analyzer, Miltenyi Biotec). The percentage of cells at each stage of the cell cycle was determined using the cell cycle platform (Watson Model) in FlowJo 10.5.3 (FlowJo LLC).

CNCC FACs: hESC derived CNCCs were purified from a differentiating cell population using the cranial neural cell surface markers CD266-PE and CD271-PE. CNCCs were sorted on a FACS Aria cell sorter (BD Bioscience) using BD FACSDiva software (ver: 2.0). See methods: "CNCC surface antigen fluorescence-activated cell sorting" for further details.

Transcription factor flow cytometry was performed on MACSquant Analyser (Miltenyi Biotec) and data analysed using FlowJo 10.5.3 (FlowJo

LLC). See methods: "Transcription Factor Flow Cytometry" for more details.

qPCR experiments were performed in triplicate (3 x biological replicates and 3 x technical replicates per condition) on a Bio-Rad C1000 Touch Thermal cycler PCR machine and analysed using the Bio-Rad CFX Manager Software (ver: 3.1). For qRT-PCR, expression was normalised to GAPDH using the deltadelta CT method in the Bio-Rad CFX manager software. For ChIP-qPCR, % recovery was calculated as (100*2^(adjusted input Ct-average ChIP Ct)), and enrichment was calculated vs TMCC1(a negative control for G4 formation) as previously described in the protocols paper Hansel-Hertsch et al 2018.

Western blots were performed on a Wes Protein Simple Western System (ProteinSimple) according to the manufacturer's protocol (https://proteinsimple.com/) using an anti-rabbit or anti-mouse detection module and corresponding antibodies. Bands were quantified as area-under-the-curve using Compass software (ProteinSimple, Ver: 5.0.1).

Code is available on the lab github webpage https://github.com/sblab-bioinformatics/G4_in_stem_cells_diff.

For manuscripts utilizing custom algorithms or software that are central to the research but not yet described in published literature, software must be made available to editors and reviewers. We strongly encourage code deposition in a community repository (e.g. GitHub). See the Nature Research guidelines for submitting code & software for further information.

Data

Policy information about availability of data

All manuscripts must include a data availability statement. This statement should provide the following information, where applicable:

- Accession codes, unique identifiers, or web links for publicly available datasets
- A list of figures that have associated raw data
- A description of any restrictions on data availability

The G4-ChIP-seq, ATAC-seq and RNA-seq data generated in this study have been deposited under the accession code GSE161531 [https://www.ncbi.nlm.nih.gov/geo/query/acc.cgi?acc=GSE161531]. The RNA-seq generated from the PhenDC3 differentiation experiment has been deposited under the accession code GSE166246 [https://www.ncbi.nlm.nih.gov/geo/query/acc.cgi?acc=GSE166246]. Imaging datasets are available from the corresponding author on reasonable request - the full 3D confocal images are extremely large in size. Source Data are provided with this paper. Processed data has been made available at: [https://github.com/sblab-bioinformatics/G4_in_stem_cells_diff]. The following previously published datasets were also used: GSM602296 [https://www.ncbi.nlm.nih.gov/geo/query/acc.cgi?acc=GSM602296], GSM602293 [https://www.ncbi.nlm.nih.gov/geo/query/acc.cgi?acc=GSM602293], GSM602292 [https://www.ncbi.nlm.nih.gov/geo/query/acc.cgi?acc=GSM602292], GSM602295 [https://www.ncbi.nlm.nih.gov/geo/query/acc.cgi?acc=GSM602295], ENCFF112ULZ [https://www.encodeproject.org/experiments/ENCSR972SMV/], GSM602294 [https://www.ncbi.nlm.nih.gov/geo/query/acc.cgi?acc=GSM602294], GSM2816629 [https://www.ncbi.nlm.nih.gov/geo/query/acc.cgi?acc=GSM2816629], GSM2816625 [https://www.ncbi.nlm.nih.gov/geo/query/acc.cgi?acc=GSM2816625], GSM2816627 [https://www.ncbi.nlm.nih.gov/geo/query/acc.cgi?acc=GSM2816627], GSM602291 [https://www.ncbi.nlm.nih.gov/geo/query/acc.cgi?acc=GSM602291], GSM2816619 [https://www.ncbi.nlm.nih.gov/geo/query/acc.cgi?acc=GSM2816619], GSM2816642 [https://www.ncbi.nlm.nih.gov/geo/query/acc.cgi?acc=GSM2816642], GSM2816615 [https://www.ncbi.nlm.nih.gov/geo/query/acc.cgi?acc=GSM2816615], GSM2816616 [https://www.ncbi.nlm.nih.gov/geo/query/acc.cgi?acc=GSM2816616], GSM2816631 [https://www.ncbi.nlm.nih.gov/geo/query/acc.cgi?acc=GSM2816631], GSM2816621 [https://www.ncbi.nlm.nih.gov/geo/query/acc.cgi?acc=GSM2816621], GSE86821 [https://www.ncbi.nlm.nih.gov/geo/query/acc.cgi?acc=GSE86821], GSM1817179 [https://www.ncbi.nlm.nih.gov/geo/query/acc.cgi?acc=GSM1817179], GSM1817174 [https://www.ncbi.nlm.nih.gov/geo/query/acc.cgi?acc=GSM1817174], GSM1817175 [https://www.ncbi.nlm.nih.gov/geo/query/acc.cgi?acc=GSM1817175], GSM1817176 [https://www.ncbi.nlm.nih.gov/geo/query/acc.cgi?acc=GSM1817176], GSM1817222 [https://www.ncbi.nlm.nih.gov/geo/query/acc.cgi?acc=GSM1817222], GSM1817170 [https://www.ncbi.nlm.nih.gov/geo/query/acc.cgi?acc=GSM1817170], GSM1817151 [https://www.ncbi.nlm.nih.gov/geo/query/acc.cgi?acc=GSM1817151], GSM1817152 [https://www.ncbi.nlm.nih.gov/geo/query/acc.cgi?acc=GSM1817152], GSM1817153 [https://www.ncbi.nlm.nih.gov/geo/query/acc.cgi?acc=GSM1817153], GSM1817190 [https://www.ncbi.nlm.nih.gov/geo/query/acc.cgi?acc=GSM1817190], GSM1817197 [https://www.ncbi.nlm.nih.gov/geo/query/acc.cgi?acc=GSM1817197], GSM1817181 [https://www.ncbi.nlm.nih.gov/geo/query/acc.cgi?acc=GSM1817181], GSM818033 [https://www.ncbi.nlm.nih.gov/geo/query/acc.cgi?acc=GSM818033], GSM818032 [https://www.ncbi.nlm.nih.gov/geo/query/acc.cgi?acc=GSM818032], GSM767350 [https://www.ncbi.nlm.nih.gov/geo/query/acc.cgi?acc=GSM767350], GSM767351 [https://www.ncbi.nlm.nih.gov/geo/query/acc.cgi?acc=GSM767351], GSM767355 [https://www.ncbi.nlm.nih.gov/geo/query/acc.cgi?acc=GSM767355], GSM767356 [https://www.ncbi.nlm.nih.gov/geo/query/acc.cgi?acc=GSM767356], GSM602303 [https://www.ncbi.nlm.nih.gov/geo/query/acc.cgi?acc=GSM602303], ENCFF452NFM [https://www.encodeproject.org/experiments/ENCSR800IIW/], GSE86189 [https://www.ncbi.nlm.nih.gov/geo/query/acc.cgi?acc=GSE86189]. Code and summary data is available on the lab github webpage https://github.com/sblab-bioinformatics/G4_in_stem_cells_diff.

Field-specific reporting

Please select the one below that is the best fit for your research. If you are not sure, read the appropriate sections before making your selection.

[x] Life sciences [ ] Behavioural & social sciences [ ] Ecological, evolutionary & environmental sciences

For a reference copy of the document with all sections, see nature.com/documents/nr-reporting-summary-flat.pdf

Life sciences study design

All studies must disclose on these points even when the disclosure is negative.

Sample size	Sample sizes were determined based on the standard sample sizes that are widely used in the field for each type of experiment. For example: Hansel-Hertsch et al. Nature Genetics 2016, Hansel-Hertsch et al. Nature Genetics 2020, Prescott et al. Cell 2015, Rada-Igelesias et al. Nature 2011 and Marchetti et al Journal of Medicinal Chemistry 2018. No statistical method was used to predetermine sample size.
Data exclusions	No data were excluded from experiments presented in this manuscript.
Replication	All experiments obtained results that were reproduced typically on a minimum of three independently obtained samples. E.g. in the ChIP-seq

	experiments each biological replicate was obtained from a different cell passage or after a separate cellular differentiation. For each biological replicate, three technical replicates were performed. Where this diverges, this is noted in figure legends and the Statistics and Reproducibility section of the Methods.
Randomization	The nature and timing of cultures and their treatments, as well as downstream biological processing of material did not allow for randomisation samples into experimental groups. For ChIP-seq, ATAC-seq and RNA-seq samples each biological replicate were processed together on different days over 1 year window with random sample order. For in vitro experiments, all experiments was done in a 'randomised' fashion whereby culture dishes containing the same cell population were chosen at random for experimental perturbation (e.g. differentiation or G4 ligand treatment). The use of independent biological replicates for each sample provided robust control of the data.
Blinding	The small number of samples and harvesting of samples at different time points over many months/years precluded blinding. The use of independent biological replicates for each sample provided robust repetition of data outcomes.

Reporting for specific materials, systems and methods

We require information from authors about some types of materials, experimental systems and methods used in many studies. Here, indicate whether each material, system or method listed is relevant to your study. If you are not sure if a list item applies to your research, read the appropriate section before selecting a response.

Materials & experimental systems

n/a	Involved in the study	
☐	☒	Antibodies
☐	☒	Eukaryotic cell lines
☒	☐	Palaeontology and archaeology
☒	☐	Animals and other organisms
☒	☐	Human research participants
☒	☐	Clinical data
☒	☐	Dual use research of concern

Methods

n/a	Involved in the study	
☐	☒	ChIP-seq
☐	☒	Flow cytometry
☒	☐	MRI-based neuroimaging

Antibodies

Antibodies used

CD266-PE (FN14) Antibody (Clone ITEM-4), anti-humna/mouse, Miltenyi Biotec FACS (dilution as per manufactures instructions), cat. # 130104329
CD271-PE Vivo 770 (p75NTR) Antibody (Clone ME20.4-1.H4), anti-human, Miltenyi Biotec FACs (dilution as per manufactures instructions), cat. # 130113984
BG4 ChIP-seq (see methods) IF 50nM, made-in-house as per methods
DYKDDDDK Tag Antibody (Binds to same epitope as Sigma's Anti-FLAG M2 Antibody) Cell Signalling Technologies IF 1:800, cat. # 2368
Ki67 (Clone 8D5), Cell Signaling Technology FACs 1:400, cat. # 9449
NANOG (Clone 1E6C4), Cell Signaling Technology IF 1:1000, FACS 1:1600, cat. # 4893
NESTIN (Clone 10C2), ThermoFisher, IF 1:500, cat. # MA1-110
NR2F1 ThermoFisher, IF 1:200, cat. # PA5-21611
OCT4 Cell Signaling Technology IF 1:200, FACS 1:200, cat. # 2750
OCT4 (Clone GT486), Abcam IF 1:500, cat. # ab184665
PAX6 Abcam IF 1:50, FACs 1:50, cat. # ab5790
PAX7 ThermoFisher IF 1:200, cat. # PA1-117
Phosphor-Histone H2A.X (Ser139) (Clone JBW301), Merck IF 1:200, cat. # 05-636
Anti-p75 NGF Receptor antibody (Clone NGFR5), Abcam 1:250, cat. # ab3125
SOX1 R&D Systems IF 1:150, cat. # AF3369
SOX2 (Clone 9-9-3), Abcam IF 1:200, cat. # ab79351
SOX2 (Clone 245610), R&D Systems FACs 1:200, cat. # MAB2018
SOX10 R&D Systems IF 1:200, cat. # AF2864
SSEA-4 (Clone MC-813-70), StemCell Technology IF 1:100, cat. #60062
anti-AP-2alpha Antibody (Clone 3B5), Santa Cruz IF 1:100, cat. # sc-12726
CHK1 Proteintech, WB 1:500, cat #25887-1-AP
Phospho-Chk1 (Ser345), Cell Signaling Technology, WB 1:50, cat #2341
CHK2 Proteintech, WB 1:250, cat #13954-1-AP
Phospho-Chk2 (Thr68) (C13C1) mAb, Cell Signaling, WB 1:250, cat #2197
Phospho-53BP1 (Ser1778), Cell Signaling Technology, IF, 1:300, cat #2675
GAPDH (Clone D16H11) XP mAb, Cell Signaling Technology, WB 1:50, cat #5174S
Donkey anti-mouse IgG (H+L) Alexa Fluor 488 conjugated ThermoFisher IF (1:500), cat. #A32766
Donkey anti-rabbit IgG (H+L) Alexa Fluor 594 conjugated ThermoFisher IF (1:500), cat. # A21207
Donkey anti-goat IgG (H+L) Alexa Fluor 647 conjugated ThermoFisher IF (1:500), cat. # A32849
Goat anti-mouse IgG (H+L) Alexa Fluor 488 conjugated ThermoFisher IF and FACs (1:500), cat. # A32723
Goat anti-mouse IgG (H+L) Alexa Fluor 647 conjugated ThermoFisher IF (1:500), cat. # A21236
Goat anti-rabbit IgG (H+L) Alexa Fluor 647 conjugated ThermoFisher IF and FACs (1:500), cat. # A32733
Goat anti-rabbit IgG (H+L) Alexa Fluor 488 conjugated ThermoFisher IF (1:500), cat. # A11034

Validation	For all antibodies, validation was by western blot and/or immunofluorescence and provided by the manufacturer with exception to the G4-specific antibody BG4. BG4 was developed in the Balasubramanian lab (Biffi et al. Nature Chemistry 2013: https://www.ncbi.nlm.nih.gov/pmc/articles/PMC3622242/) and prepared and validated by K.Z. Preparation of BG4 was performed according to Biffi et al. Nature Chemistry 2013 and validated using methods described in Biffi et al. Nature Chemistry 2013 and Hansel-Hertsch et al. Nature Protocols 2018 (https://pubmed.ncbi.nlm.nih.gov/29470465/).<br><br>Further validation of commercial antibodies used in this paper was performed via IF and flow cytometry. E.g. positive staining for OCT4 in hESC and loss upon differentiation to NSC and CNCC. See methods and Supplementary Fig. 1 for further details.<br>Additional validation by the manufacturer was provided for the following antibodies:<br><br>NESTIN (Clone 10C2), ThermoFisher, IF 1:500, cat. # MA1-110: "This Antibody was verified by Relative expression to ensure that the antibody binds to the antigen stated. The specificity of anti-Nestin monoclonal antibody (Product # MA1-110) was demonstrated in western blot analysis by the detection of endogenous Nestin protein expression in human neural stem cells, but not in negative control line HepG2 which is not known to express Nestin protein."<br><br>OCT4 Cell Signaling Technologies IF 1:200, FACS 1:200, cat. # 2750: "This antibody has been validated using SimpleChIP® Enzymatic Chromatin IP Kits."<br><br>SSEA-4 (Clone MC-813-70), StemCell Technologies IF 1:100, cat. #60062: "This antibody clone has been verified for labeling human ES and iPS cells grown in TeSRTM-E8TM (Catalog #05940), mTeSRTM1 (Catalog #85850), and TeSRTM2 (Catalog #05860)"<br><br>CD266-PE (FN14) Antibody (Clone ITEM-4) and CD271-PE Vivo 770 (p75NTR) Antibody (Clone ME20.4-1.H4 have additionally been validated via flow cytometry in Prescott 2015 (https://pubmed.ncbi.nlm.nih.gov/26365491/)

Eukaryotic cell lines

Policy information about cell lines

Cell line source(s)	H9 (WA09), H1 (WA01) and H1 OCT4-EFGP (modified WA01) human embryonic stem cells (hESCs) and H9-derived neural stem cells (NSC) were purchased from WiCell, USA. Cranial neural crest cells (CNCCs) were generated in this study from WA09 as previously described in Prescott et al, 2015. hESCs work was authorised by the Steering Committee for the UK Stem Cell Bank and for Use of Stem Cells (MRC).
Authentication	hESC cell lines were authenticated by STR genotyping conducted in-house by the CRUK CI Research and Instrumentation and Cell Services (initial authentication of genotypes was performed by WiCell) and by flow cytometry and immunofluorescence for markers of pluripotency and loss of expression of proteins upon differentiation. NSC and CNCCs were validated by flow cytometry and immunofluorescence for markers unique to cell lineages and by RNA-seq and comparing to equivalent cell types generated in Xie et al 2013 and Prescott et al 2015 as outlined in the paper.
Mycoplasma contamination	All cell lines used were routinely tested for mycoplasma and tested negative, as part of the CRUK CI institute policy
Commonly misidentified lines<br>(See ICLAC register)	No commonly misidentified cell lines were used in this study.

ChIP-seq

Data deposition

☒ Confirm that both raw and final processed data have been deposited in a public database such as GEO.

☒ Confirm that you have deposited or provided access to graph files (e.g. BED files) for the called peaks.

Data access links<br>*May remain private before publication.*	https://www.ncbi.nlm.nih.gov/geo/query/acc.cgi?acc=GSE161531
Files in database submission	62 fastq files corresponding to 62 samples profiled. Specifically: 28 IP samples and I input samples correspond to G4-ChIP-seq profiling; 9 samples correspond to ATAC-seq; 16 samples correspond to RNA-seq. For each G4-ChIP-seq and ATAC-seq library, a peak file with local enrichments has been submitted together with the consensus regions across multiple technical replicates and biological replicates. For the each of the RNA-seq library, gene counts estimated with Rsem have been submitted. As a supplementary file, the matrix with each individual expression values (TPM, transcript per million) have been submitted.
Genome browser session<br>(e.g. UCSC)	No longer applicable.

Methodology

Replicates	For each cell line, 3 biological replicates have been profiled by ATAC-seq and G4-ChIP-seq. For each biological replicate, in the case of G4-ChIP-seq, 3 technical replicates and 1 corresponding input sample have been profiled. RNA-seq: 5 biological replicates for ESC (biological replicate 4 was screened with 4 technical replicates and biological replicate 5 with 2 technical replicates); 3 biological replicates for CNCC cells; 4 biological replicates for NSC.
Sequencing depth	The number of not-duplicated single-end sequencing reads (NextSeq 500) for G4-ChIP-seq was around 30M reads across all samples.

	RNA-seq libraries (single-end sequenced with HiSeq 4000) sequencing depth was on average 26M reads for hESC, CNCC and NSC cell lines and 22M reads for the PhenDC3 differentiation experiment. ATAC-seq libraries sequencing depth was in average around 150M reads (paired-end, NextSeq 500)
Antibodies	The scFV BG4 was developed in the Balasubramanian lab (Biffi et al. Nature Chemistry 2013) and prepared and validated by K.Z. in-house. Preparation was performed according to Biffi et al. Nature Chemistry 2013 and validated using methods described in Biffi et al. Nature Chemistry 2013 and Hansel-Hertsch et al. Nature Protocols 2018.
Peak calling parameters	G4-ChIP-seq peaks have been called using MACS2 with default qvalue (minimum FDR of 0.05), default human size and --broad option. ATAC-seq peaks have been called with macs2 default options.
Data quality	Only enrichment regions with FDR below 0.05 were considered in this study.

Software

Software, tools and environment used

Step Software name and version
fastqc : FastQC v0.11.7
adapter trimming: cutadapt version 1.16
alignment: bwa mem 0.7.17-r1188
duplicate marking: picard-2.20.3
bam file indexing, sorting and handeling : samtools Version: 1.8 (using htslib 1.8)
bigWig track generation: bamCoverage 3.3.0 (deepTools 3.3.0)
peak calling : macs2 2.1.2
bed files processing and manipulation: bedtools v2.27.1
System info:
Linux kernel version 3.10.0-1127.18.2.el7.x86_64
cluster management and job scheduling system slurm 20.02.4

R session info:
print(sessionInfo())
R version 3.6.1 (2019-07-05)
Platform: x86_64-apple-darwin15.6.0 (64-bit)
Running under: macOS High Sierra 10.13.6
Matrix products: default
BLAS: /System/Library/Frameworks/Accelerate.framework/Versions/A/Frameworks/vecLib.framework/Versions/A/libBLAS.dylib
LAPACK: /Library/Frameworks/R.framework/Versions/3.6/Resources/lib/libRlapack.dylib

locale:
[1] en_GB.UTF-8/en_GB.UTF-8/en_GB.UTF-8/C/en_GB.UTF-8/en_GB.UTF-8

attached base packages:
[1] grid stats graphics grDevices utils datasets methods base

other attached packages:
[1] ComplexHeatmap_2.0.0 dplyr_1.0.2 edgeR_3.26.8 limma_3.40.6

loaded via a namespace (and not attached):
[1] Rcpp_1.0.5 pillar_1.4.6 compiler_3.6.1 RColorBrewer_1.1-2 tools_3.6.1 digest_0.6.25 evaluate_0.14
[8] lifecycle_0.2.0 tibble_3.0.3 lattice_0.20-41 clue_0.3-57 pkgconfig_2.0.3 png_0.1-7 rlang_0.4.7
[15] rstudioapi_0.11 yaml_2.2.1 parallel_3.6.1 xfun_0.17 knitr_1.29 cluster_2.1.0 generics_0.0.2
[22] GlobalOptions_0.1.2 vctrs_0.3.4 locfit_1.5-9.4 tidyselect_1.1.0 glue_1.4.2 R6_2.4.1 GetoptLong_1.0.2
[29] rmarkdown_2.3 purrr_0.3.4 magrittr_1.5 ellipsis_0.3.1 htmltools_0.5.0 splines_3.6.1 shape_1.4.5
[36] circlize_0.4.10 colorspace_1.4-1 crayon_1.3.4 rjson_0.2.20

Processing code and scripts are available at: https://github.com/sblab-bioinformatics/G4_in_stem_cells_diff.

Flow Cytometry

Plots

Confirm that:

- [x] The axis labels state the marker and fluorochrome used (e.g. CD4-FITC).
- [x] The axis scales are clearly visible. Include numbers along axes only for bottom left plot of group (a 'group' is an analysis of identical markers).
- [x] All plots are contour plots with outliers or pseudocolor plots.
- [x] A numerical value for number of cells or percentage (with statistics) is provided.

Methodology

Sample preparation	Samples were obtained and treated as described in methods section. To asses DNA content, hESCs, CNCCs and NSCs grown in culture were dissociated with StemPro Accutase and fixed with 4 % paraformaldehyde and permeabilised with 0.25% (v/v) Triton-X prior to staining with DAPI. For transcription factor flow cytometry, cells were fixed with 4 % paraformaldehyde and permeabilised with PBS/0.7% (v/v) Tween20 and blocked with 1% (w/v) BSA , 10% normal goat serum, 0.5% Tween20 in PBS prior to staining with primary antibodies as per protocol in Turac et al PLoS One, 2013. For OCT4-EGFP monitoring, H1-hESC OCT4-EGFP cells were washed twice with DPB, dissociated with StemPro Accutase and resuspended in neural induction media prior to flow cytometry analysis. All samples were passed through a 70 um filter.
Instrument	MACSquant VYB Analyzer (Miltenyi Biotec) and FACS Aria cell sorter (BD Bioscience) were used for data acquisition.
Software	FlowJo v10.5.3 (FlowJo LLC)) was used for flow cytometry analysis.
Cell population abundance	Minimum of 10,000 cells were recorded. See methods for further details.
Gating strategy	For gating single cells: Cells were gated using Forward Scatter Area (FSC-A) vs Side Scatter Area (SSC-A) to remove debris. Single cells were then gated from doublets/clumps using FSC-A vs FSC-H ratio and gating of objects with FSC-A:FSC-H ratio of roughly 1. For gating cells with positive expression of marker of interest, a secondary antibody control cell population was used. Gating strategy example in Supplementary Fig. 1g and 3e.

☒ Tick this box to confirm that a figure exemplifying the gating strategy is provided in the Supplementary Information.
