# Supplementary figures and images for: G-quadruplex DNA structures in human stem cells and differentiation
Source: Nat Commun. 2022 Jan 10;13:142. doi: 10.1038/s41467-021-27719-1 (PMC8748810; doi:10.1038/s41467-021-27719-1)

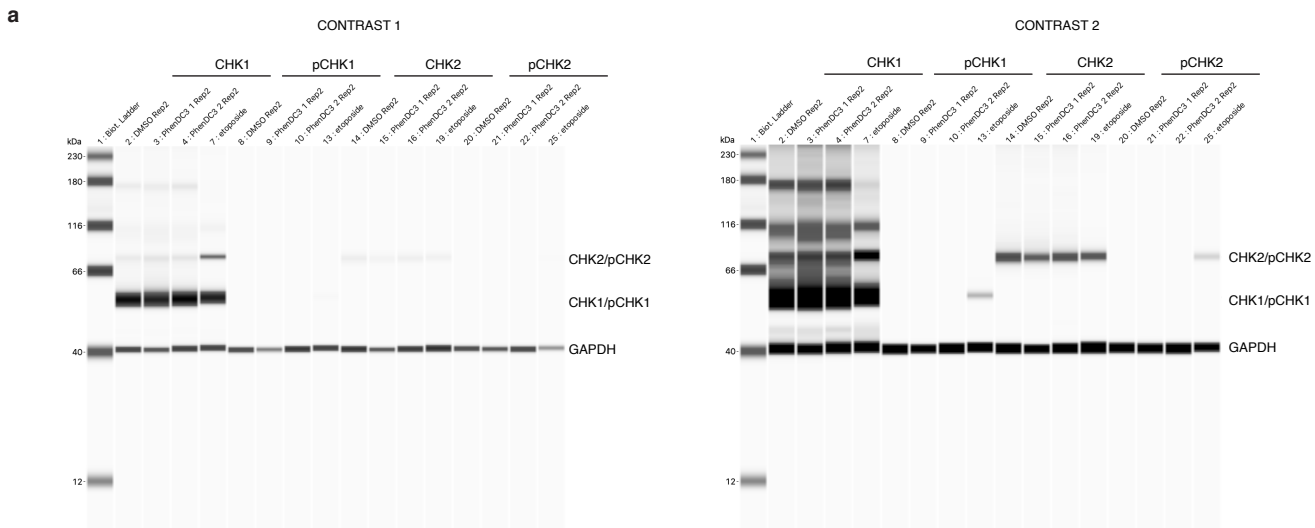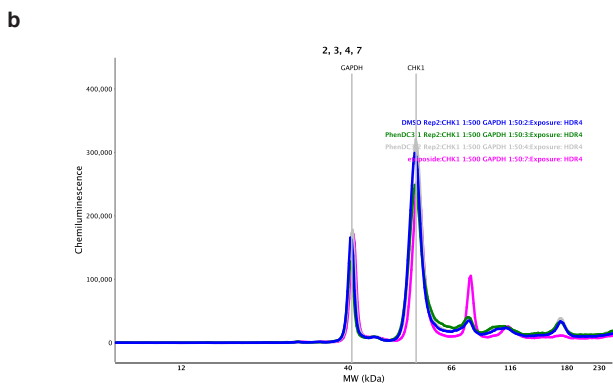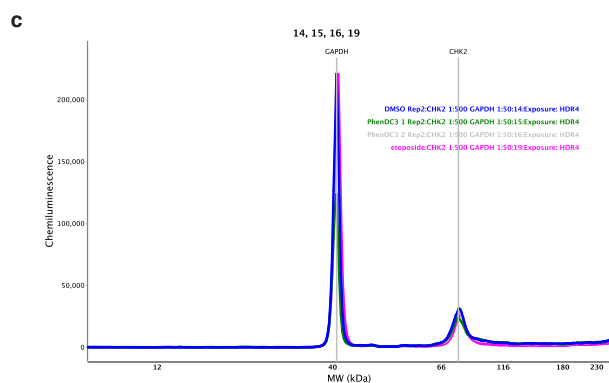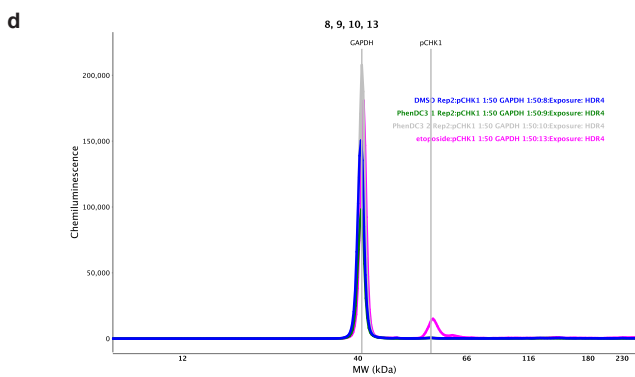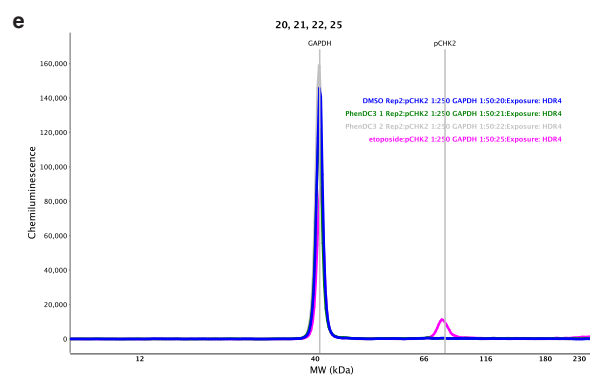

Supplement: Supplementary file 9 — Source Data [file 41467_2021_27719_MOESM9_ESM.zip › Source data fig.pdf]
